# Selective targeting of coagulation factor X Gla domain by negatively charged gold nanoparticles: a novel method for controlled antithrombotic therapy

**DOI:** 10.1016/j.mtbio.2025.102378

**Published:** 2025-10-01

**Authors:** Shixin Li, Yuye Yin, Dongmei Hou, Yongchao Jin, Yuan Zhao, Jiangbo Tong, Xu Liu, Guomin Shen, Tongtao Yue, Kang Liu, Yi Gu, Luju Chen, Fangzhe Ren, Jinlin Huang, Jian-Ke Tie, Zhenyu Hao

**Affiliations:** a College of Bioscience and Biotechnology, Yangzhou University, Yangzhou, 225009, China; b College of Chemistry and Chemical Engineering, Yangzhou University, Yangzhou, 225009, China; c Department of Cell Biology, School of Basic Medical Sciences, Harbin Medical University, Harbin, 150081, China; d Institute of Coastal Environmental Pollution Control, Key Laboratory of Marine Environment and Ecology, Ministry of Education, Ocean University of China, Qingdao, Shandong, 266100, China; e Laboratory for Marine Ecology and Environmental Science, Qingdao National Laboratory for Marine Science and Technology, Qingdao, Shandong, 266237, China; f Joint International Research Laboratory of Agriculture and Agri-Product Safety, Ministry of Education of China, Yangzhou, Jiangsu, 225009, China; g Department of Biology, The University of North Carolina at Chapel Hill, Chapel Hill, NC, 27599, USA; h Affiliated Hospital, Yangzhou University, Yangzhou, Jiangsu, 225009, China

**Keywords:** Venous thromboembolism, Gla domain, Coagulation factor X, Antithrombotic strategy

## Abstract

Venous thromboembolism (VTE) presents a significant global health burden due to its high incidence and potentially life-threatening complications. Although anticoagulants targeting vitamin K-dependent (VKD) factors, particularly factor X (FX), are widely employed, their efficacy is often limited by bleeding risks arising from off-target effects. Nanoparticle-based strategies, by contrast, enable precise and tunable modulation of protein activity through controlled adjustments in particle size, charge, and functionalization. In this work, we engineered negatively charged gold nanoparticles (GNPs) of defined sizes to selectively interact with the γ-carboxyglutamic acid (Gla) domain of VKD coagulation proteins. Using computational simulations, we systematically compared their binding conformations and affinities between GNPs and diverse VKD coagulation proteins, uncovering a size-dependent binding mechanism. This finding was subsequently validated through biochemical assays at both the molecular and cellular levels. Notably, GNPs with diameters of 2–3 nm demonstrated significantly higher affinity for FX compared to other VKD proteins, such as factor IX and protein C. This specific binding triggered substantial conformational changes in FX, diminishing its membrane-binding affinity. These structural alterations also reduced its enzymatic activity and impaired its activation efficiency within the coagulation cascade, thereby effectively attenuating the cascade by selectively modulating FX activity. Comprehensive *in vitro* coagulation assays and *in vivo* murine thrombosis models further validated that GNP treatment effectively prolonged coagulation time, demonstrating robust antithrombotic efficacy. Collectively, our results establish a novel nanoparticle-based therapeutic paradigm for targeting FX, offering an innovative and promising approach for enhancing the safety and efficacy of VTE prevention and management.

## Highlights

• GNPs selectively bind VKDP Gla domain in a size-dependent manner.
• Negatively charged GNPs (2–3 nm) exhibit strong affinity for FX.
• GNPs induce FX conformational changes, disrupting coagulation activity.
• GNPs prolong clotting time *in vitro* and *in vivo*, offering novel VTE therapy.

## Graphical abstract

**Figure:**
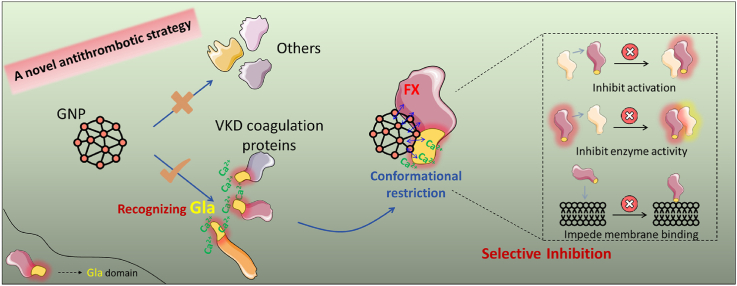

## 1 Introduction

Venous thromboembolism (VTE), comprising deep vein thrombosis (DVT) of the lower limbs with or without pulmonary embolism, affects approximately 1–2 individuals per 1,000 annually, particularly in developed countries [1,2]. The impact of venous thrombosis extends well beyond acute complications, such as pulmonary embolism (PE), and includes chronic conditions like post-thrombotic syndrome (PTS) [1,3]. The high incidence of VTE underscores the critical need for effective prevention and early intervention [4]. Key challenges in managing venous thrombosis include the difficulty of early diagnosis, the increased risk of bleeding associated with treatment, and the complexities of long-term patient management [5,6].

Elevated plasma levels of coagulation factors, prompting an intensified coagulation cascade, are a major pathogenic mechanism in VTE, alongside hereditary predispositions [7,8]. This hypercoagulable state plays a crucial role in the initiation and progression of VTE, significantly exacerbating the risk of thrombotic events. Consequently, targeted anticoagulant therapies that inhibit specific coagulation factors have become essential strategies in both the prevention and management of VTE [9,10]. Among the most important targets are the vitamin K-dependent (VKD) coagulation factors, including factors II, VII, IX, and X, which are integral components of the coagulation cascade [11]. Modulating these factors is vital for managing hypercoagulability and preventing thrombotic complications [12,13]. Historically, warfarin, as a vitamin K antagonist, has been widely employed by inhibiting the vitamin K cycle, thus reducing VKD coagulation factor activity, alleviating symptoms, and improving patient outcomes [14]. However, long-term use of warfarin is associated with several adverse effects and requires regular monitoring of the International Normalized Ratio (INR) to ensure therapeutic efficacy and minimize the risk of complications [15]. In light of these limitations, small-molecule agents that specifically target coagulation factors such as Factor X and Factor II, including direct Factor Xa inhibitors and low-molecular-weight heparins [16], represent integral strategies in the prevention and management of VTE [9,17]. Coagulation Factor X (FX) plays a pivotal role in the convergence of both the intrinsic and extrinsic coagulation pathways, making its inhibitors, such as rivaroxaban and apixaban, highly effective due to their potent anticoagulant activity and specificity [18]. However, these treatments are not without limitations, they can occasionally cause off-target effects due to interactions with other serine proteases, leading to rare but serious adverse events, including gastrointestinal bleeding or pulmonary embolism [19,20].

Compared to small-molecule drugs, nanoparticles offer distinct advantages due to their controllable size and surface properties, enabling them to specifically interact with proteins and modulate their biological functions [[21], [22], [23]]. Engineered nanoparticles have been designed to specifically target key proteins involved in the blood coagulation cascade, with their physicochemical properties critically influencing these interactions [[24], [25], [26]]. For instance, surface modifications and adjustments in negative charge density of zeolite can influence the adsorption states of Factor Va, shifting between single-foot and double-foot configurations [27]. Similarly, interaction modes of coagulation Factor XII (FXII) can be regulated by nanoparticles composition and size, resulting in structural alterations and activation [28]. Furthermore, modifications of chiral ligands have been shown to influence their interactions with blood proteins [29]. Given the critical role and specific structure features of FX, designing targeted nanoparticles to selectively modulate its structure and function holds significant therapeutic potential for preventing thrombotic events.

VKD coagulation proteins, including coagulation factors II (FII), VII, IX, X, and anticoagulants C, S, and Z, possess a unique γ-carboxyglutamic acid (Gla) domain [30,31]. Gamma-glutamyl carboxylation of the Gla domain is essential for their function, enabling Ca^2+^ binding and interaction with negatively charged membrane surfaces during the coagulation cascade [32]. We hypothesize that negatively charged nanoparticles can effectively target and recruit the carboxylated Gla domains of VKD coagulation factors, thereby selectively modulating their activity and providing potential therapeutic benefits for antithrombotic therapy. To explore this approach, we selected gold nanoparticles (GNPs) as model materials, which have been widely utilized in biomedical applications, including drug delivery, imaging, and therapy, due to their advantages of biocompatibility and ease of functionalization [33,34].

In our work, we synthesized negatively charged GNPs of varying sizes to selectively target the Gla domain and modulate FX activity. Both simulations and experiments demonstrated that interactions between GNPs and VKD coagulation proteins are significantly influenced by particle size. Specifically, GNPs with a diameter of 2–3 nm showed stronger binding to FX than coagulation factor IX (FIX) or protein C (PC). GNP in this size range induced notable conformational changes in FX by immobilizing its Gla and functional domains, thus reducing its affinity for lipid membranes and impairing its activation efficiency and enzymatic activity. Both *in vitro* and *in vivo* coagulation assays demonstrated that GNPs significantly prolonged clotting time, correlating with their potent antithrombotic efficacy *in vivo*. This work presents a novel strategy for the treatment and prevention of VTE.

## 2 Materials and methods

### 2.1 Materials and cell lines

Materials used include HAuCl_4_, PVP (polyvinylpyrrolidone, K-30, 40 kDa), NaBH_4_, trisodium citrate, mercaptobutyric acid (Aladdin, Shanghai, China), vitamin K1 (MedChemExpress, NJ, USA), Cell Counting Kit-8, thrombin, chromogenic substrate S2238 (Boatman, Shanghai, China), Pefachrome FIXa (Pentapharm DSM, Basel, Switzerland), aspartate aminotransferase (AST) and alanine aminotransferase (ALT) activity assay kits (Elabscience, Wuhan, China), Xfect transfection reagent (Takara, CA, USA), liposomes (Coag Reagent I, Avanti, AL, USA), mammalian expression vector pBudCE4.1 and pcDNA3.1 Hygro(+) (Invitrogen, CA, USA), mouse anti-carboxylated FXgla domain monoclonal antibody, donkey anti-rabbit IgG (H + L) highly cross-adsorbed secondary antibody Alexa Fluor Plus 647 and goat anti-mouse IgG (H + L) secondary antibody HRP (Thermo Fisher, CA, USA), HRP-conjugated rabbit anti-His-Tag monoclonal antibody, HRP-conjugated goat anti-rabbit IgG (H + L) and rabbit anti His-tag mAb (Abclonal Biotechnology, Wuhan, China), Factor X rabbit mAb (Zenbio, Chengdu, China), sheep anti-human Factor IX affinity-purified IgG (Affinity Biologicals, Canada), anti-HSA monoclonal antibody (Proteintech, Wuhan, China), mAb against γ-Carboxyglutamyl residues (BIOMEDICA, DE, USA), activated partial thromboplastin time kit (APTT) and supply prothrombin time (PT) assay kit (Succeeder, Beijing, China), human standard plasma, FX-deficient human plasma (SIEMENS, Germany), Cleaved-F10 (A41) antibody and human activated coagulation factor X (FXa) ELISA kit (CUSABIO, Wuhan, China), and RVV-X Activator (Prolytix, VT, USA). HEK293 (RRID:CVCL_0045) and HEK293T (RRID:CVCL_0063) cell lines, widely used for their high transfection efficiency and versatility in gene expression studies, were selected for this study. Both cell lines were originally obtained from ATCC (VA, USA) and maintained under standard laboratory conditions as of June 2022. Prior to use, the cells were authenticated and confirmed to be free of contamination.

### 2.2 DNA manipulations and plasmid constructions

Mammalian expression vector pcDNA3.1 Hygro (+), with the cDNA of *FIX, FX, PC*, as well as *FIXfullHis*, *FXfullHis*, *FIXMBPHis*, and *FXMBPHis* were used for cloning and expression, as previously described [35]. *FIXfullHis* and *FXfullHis* indicated that a His-tag was added to *FIX* and *FX*, respectively. *FIXMBPHis* and *FXMBPHis* represent the constructs in which the functional domains of *FIXfullHis* and *FXfullHis* were replaced with that of *maltose-binding protein* (*MBP*), respectively. Additionally, *FX, FIX and FII* lacking the Gla domain (*FXdegla, FIXdegla and FIIdegla*) was cloned into the expression vector. All construct sequences were verified by DNA sequencing at Genewiz Inc. (Suzhou, China).

### 2.3 Theoretical calculations

We employed molecular docking and molecular dynamics simulations to investigate the binding mode of rivaroxaban-FXa, the interactions between VKD coagulation proteins and various GNPs, as well as the subsequent effects on membrane binding. All molecular docking simulations were performed using AutoDock Vina [36] (version 1.1.2), while molecular dynamics simulations were carried out with GROMACS 2019-3 [37]. Detailed simulation parameters can be found in the Supplemental Materials.

### 2.4 Binding kinetics of VKD coagulation proteins with GNPs and secondary structure analysis

Three GNPs with different diameters (<2 nm, 2–3 nm, and > 8 nm) were synthesized based on the established protocols [[38], [39], [40]]. Mercaptobutyric acid was introduced to impart negative surface charges. Interactions between GNPs and VKD proteins (FIX, FX, and PC) were quantified using isothermal titration calorimetry (ITC) (Nano ITC, TA Instruments, USA). Circular dichroism (CD, Jasco J-810, Japan) spectroscopy was employed to examine the impact of GNPs on the structural changing of proteins by recording the far-UV region (190–280 nm) [41].

### 2.5 Fluorescence colocalization assay

To validate the subcellular targeting specificity of FX, HEK293T cells transiently expressing VKD coagulation proteins (including FX, FXdegla, FIX, FIXdegla, FII, FIIdegla, and PC) underwent nanoparticle-based colocalization assays [42]. Cells were incubated with 2-3 nm GNPs at 37 °C for 1 h. Samples were sequentially treated with primary (Rabbit-anti His-tag mAb) and secondary antibodies (Donkey anti-Rabbit IgG (H + L) Highly Cross-Adsorbed Secondary Antibody, Alexa Fluor Plus 647). Fluorescent signals were captured using an inverted fluorescence microscope (Olympus IX73, Japan) after mounting coverslips with ProLong antifade reagent.

### 2.6 Analysis of GNP-plasma protein complexes and FX targeting

GNP-protein complexes in plasma were analyzed as previously described [43]. FX-deficient plasma was clarified by centrifugation and sequential filtration (0.45 μm and 0.22 μm) before incubation with GNPs for 30 min, with or without FX supplementation. Samples were then ultrafiltered, and both retentates and filtrates were subjected to Western blot and ELISA. FX was detected using a rabbit monoclonal anti-FX antibody with HRP-conjugated goat anti-rabbit IgG, while FIX and HSA were probed with sheep anti-FIX and mouse anti-HSA antibodies, respectively. To assess the kinetics of complex formation, FX, FIX, and HSA levels associated with GNPs were quantified at multiple time points (0.2 min, 0.5 min, 2 min, 10min, 30 min, 60 min).

### 2.7 Measurement of FX's enzymatic activity, activation and membrane binding

FX enzyme activity with and without GNPs were assessed using a chromogenic substrate method [44]. Active FX (FXa, activated by RVV-X) was incubated with GNPs for 20 min in darkness, and FX activity was measured at 405 nm after adding Chromogenix S-2765. For comparison, thrombin and FIXa activities were quantified using the chromogenic substrates Boatman™ S-2238 and Pefachrome FIXa, respectively. For activation, FX was incubated with 2-3 nm or > 8 nm GNPs at 37 °C for 30 min. RVV-X was added to cleave FX, and the samples were incubated at 37 °C for 10 min. The samples were subsequently subjected to Western blot using Cleaved-F10 (A41) antibody. The effect of GNPs on protein-membrane binding was assessed using a protein-lipid overlay assay (PLO) [45]. Liposomes were dot-dried on a PVDF membrane and incubated with VKD coagulation proteins in blocking buffer, with or without GNPs. Membrane-bound proteins were measured with histidine affinity antibodies to evaluate GNPs' impact on the binding efficiency of VKD coagulation proteins.

### 2.8 Effect of GNPs on blood coagulation in mouse models

Blood coagulation was evaluated with mouse tail-bleeding assays [46]. Twelve BALB/c mice (7-week-old) were divided into control (100 μL, normal saline) and experimental (100 μL, 100 μM GNPs) groups. Three hours post-injection, tails were trimmed, immersed in saline, and coagulation was assessed by measuring optical density at 405 nm (OD_405 nm_). Blood plasma clotting times (APTT, PT) were evaluated in both mouse and human plasma (with or without GNPs) [46]. FXa was quantified in standard human plasma using a Human activated coagulation factor X (FXa) ELISA kit (Cusabio), while mouse plasma FXa levels were assessed with Cleaved-F10 (A41) antibody (Cusabio).

In the tail thrombosis assay, rivaroxaban (a clinically approved FXa inhibitor) was used as a positive control. A total of 36 mice were randomly assigned to four groups: (i) daily intravenous injections of GNPs (100 μL, 100 μM) for one week; (ii) daily oral administration of rivaroxaban (300 μL, 0.5 mg/mL) as reported previously [47,48]; and (iii–iv) daily intravenous injections of normal saline (100 μL) for one week. After carrageenan-induced thrombosis [49], tail thrombus length was measured. Organs and feces from 36 additional mice injected with GNPs were collected for metabolic analysis and gold content quantification via Inductively Coupled Plasma Mass Spectrometry（ICP-MS，Elan DRC-e) [50]. Liver and kidney tissues were fixed, stained with Hematoxylin and Eosin (H&E), and analyzed histologically [51].

An additional cohort of 24 mice was divided into three groups to assess bleeding safety [52]. After one week of treatment, tail transection was performed to record blood loss, bleeding time, and recurrent bleeding events. Plasma samples were collected for measurement of AST and ALT using commercial kits, alongside coagulation parameters (APTT and PT). The INR was also calculated to further evaluate bleeding risk. All animal experiments were approved by Institutional Animal Care and Use Committee (IACUC) of Yangzhou University (No.202502079), and performed according to guidelines.

## 3 Results

### 3.1 Negatively charged GNPs specifically recognize the Gla domain of VKD coagulation proteins

**Fig. 1:**
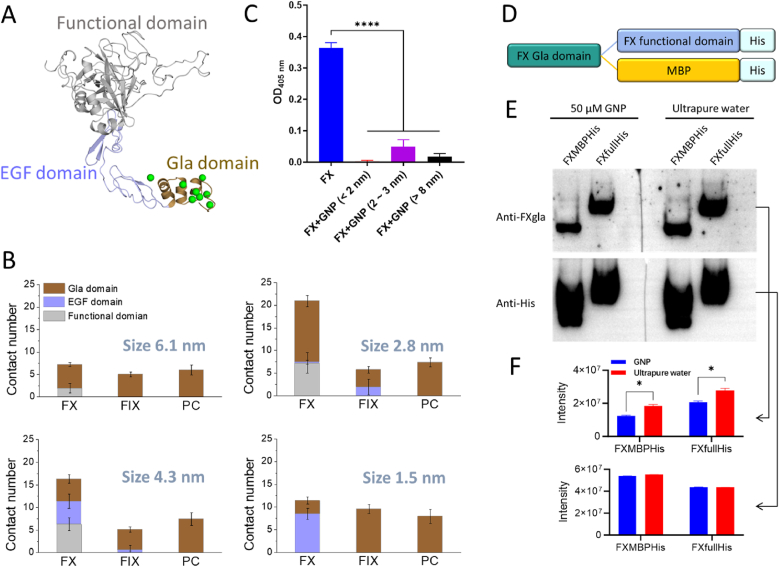
Validation of negatively charged nanoparticles specifically recognizing the Gla domain of VKD coagulation proteins. (A) Schematic representation of structural domains in VKD coagulation proteins, with Ca^2+^ shown in green. (B) Residue contact number analysis indicating interactions between structural domains of VKD coagulation proteins and GNPs of varying sizes. (C) ELISA-based assessment of the effect of GNPs on anti-FXgla antibody binding to the Gla domain of FX. (D) Topology diagram of distinct reporter proteins. (E) Western blot analysis comparing binding patterns of 2-3 nm GNPs with various reporter proteins, detected using Anti-FXgla and Anti-His antibody. (F) Quantitative analysis (ImageJ-based) of FX expression levels from Western blot bands in panel (E). ∗∗∗∗*p* < 0.0001, ∗*p* < 0.05. (For interpretation of the references to colour in this figure legend, the reader is referred to the Web version of this article.)

VKD procoagulant and anticoagulant factors are pivotal regulators of coagulation homeostasis [11]. Unlike other blood proteins, VKD factors feature a unique structural motif called Gla domain (Fig. S1 and Fig. 1A), which chelates Ca^2+^ and binds to negatively charged lipid membranes, a key requirement for their function in coagulation [32]. We hypothesized that negatively charged nanoparticles could specifically target and recruit the Gla domain of VKD factors, modulating coagulation homeostasis. To evaluate this hypothesis, we performed computational simulations to investigate interactions between structural domains of VKD factor (FX, FIX, and PC) and GNPs ranging in size from 1.5 nm to 6.1 nm (Fig. S2 and Fig.S3). The computational results revealed that the Gla domain of VKD factors plays a crucial role in GNP binding compared to EGF and functional domains, as evidenced by residue contact number (Fig. 1B) and interacting energy (Fig. S4). Over 200 ns MD simulations, GNPs of various sizes consistently interacted with Gla domains via Ca^2+^-mediated bridging, demonstrating their specific recognition of VKD coagulation proteins (Fig. S5).

To experimentally validate this Gla-recognition mechanism, we synthesized three mercaptobutyric acid-modified GNPs with distinct diameters (<2 nm, 2–3 nm, and >8 nm) (Fig. S6). The ELISA studies focusing on FX revealed significant attenuation of anti-FXgla antibody binding (OD_405nm_ reduction >50 %) upon GNP treatment, regardless of the particle size (Fig. 1C). These findings demonstrated that GNPs universally occupy the Gla domain in a size-independent manner, as evidenced by MD simulations (Fig. 1B). Moreover, we substituted the functional domains of FX with the reporter protein FXMBPHis (Fig. 1D) to further investigate the critical role in GNP binding recognition. Considering the strong interaction of 2.8 nm GNP with FX's Gla domain compared to FIX and PC (Fig. 1B), we selected 2–3 nm GNP experimentally for further investigations. Western blot analysis indicated that the Gla domain of the reporter proteins was effectively shielded by GNPs, regardless of whether the subsequent domain was the functional domain of FX (FXfullHis) or the unrelated protein MBP (FXMBPHis) (Fig. 1E and F), confirming GNPs' specificity toward the Gla domain rather than general epitope masking. Parallel experiments with FIX yielded analogous Gla-dependent inhibition patterns (Fig. S7), reinforcing the broad applicability of anionic GNPs in recognizing VKD coagulation proteins.

**Fig. 2:**
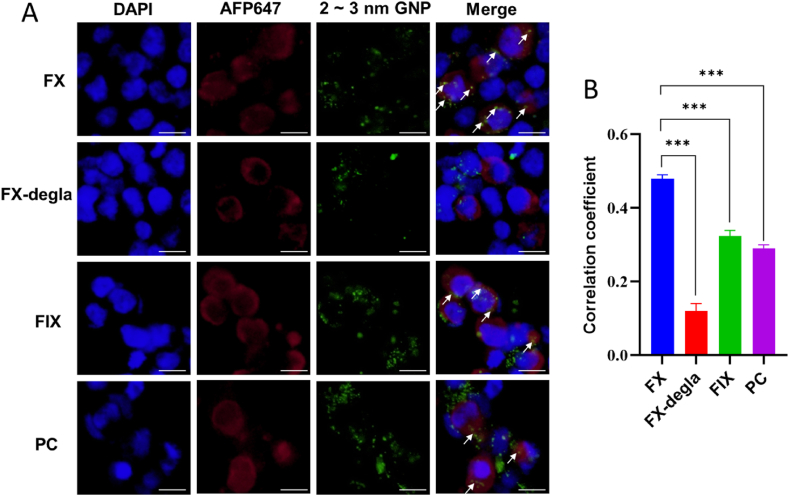
Fluorescence colocalization confirming targeted interaction of 2-3 nm GNPs with FX. (A) Representative fluorescence microscopy images showing colocalization between GNPs and VKD coagulation proteins. Cell nuclei counterstained with DAPI (blue), while VKD coagulation factors were labeled with Alexa Fluor Plus 647 (AFP647)-conjugated polyclonal antibody (red). White arrows indicate area of colocalization. The scale bar represents 10 μm. (B) Quantitative correlation analysis of fluorescence colocalization between GNPs and indicated VKD coagulation proteins, ∗∗∗*p* < 0.001. (For interpretation of the references to colour in this figure legend, the reader is referred to the Web version of this article.)

Encouraged by the demonstrated capacity of negatively charged GNPs, we further investigated the targeting efficiency of 2-3 nm GNPs in the cellular environment using fluorescence colocalization assay [42]. As shown in Fig. 2A, the colocalization of GNPs was significantly greater for full-length FX compared to FX lacking the Gla domain, further reinforcing the importance of the Gla domain in recognition. After Gla domain deletion, the correlation coefficient between GNPs and FX decreased from 0.48 to 0.12 (Fig. 2B). This Gla domain-dependent recognition was further substantiated by the markedly lower correlation coefficients observed for GNP interactions with Gla-domain-less FIX and FII (Fig. S8). Moreover, correlation coefficients for FIX and PC were substantially lower than FX despite the presence of the Gla domain in all three proteins (Fig. 2), consistent with binding strength analysis (Fig. 1B). Given the higher physiological concentration of FII, we also assessed its colocalization with GNPs. The correlation coefficient for FII was markedly lower than that for FX, further supporting the selective targeting of 2-3 nm GNPs toward FX (Fig. S8). This selective targeting toward FX indicates significant potential to regulate the coagulation cascades.

### 3.2 Size-dependent differential binding of GNPs with VKD coagulation proteins

**Fig. 3:**
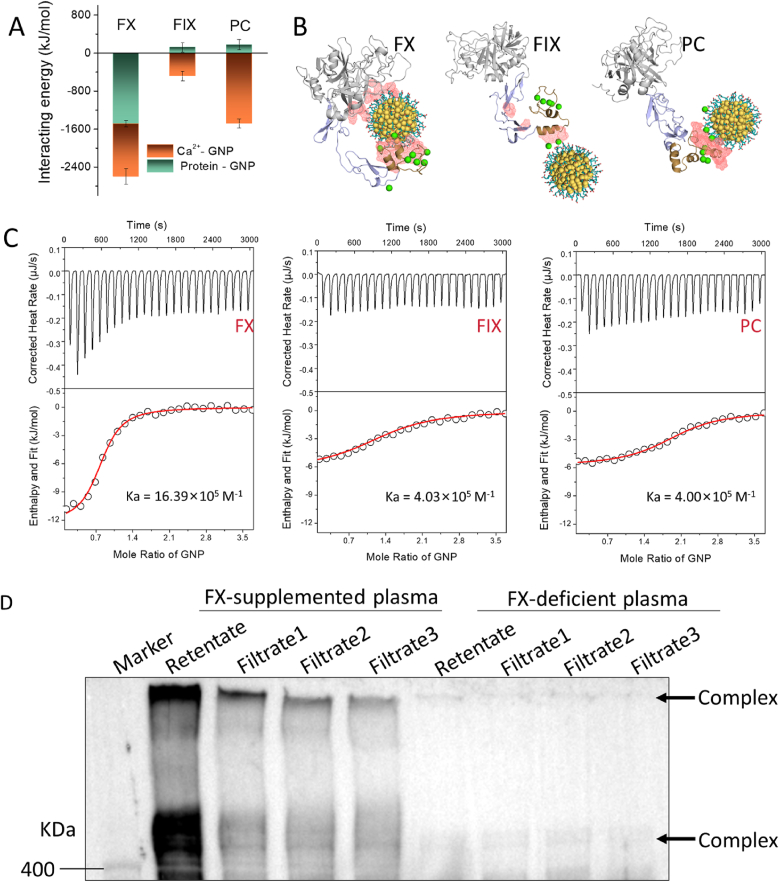
Differential interactions of GNPs with various VKD coagulation proteins. (A) Calculated interacting energies between 2.8 nm GNP and the residues of VKD coagulation proteins and their associated Ca^2+^, derived from the final 50 ns molecular simulation trajectories. (B) Representative binding conformations depicting interaction modes between 2.8 nm GNP and FX, FIX, or PC. Red network-covered areas indicate high-probability binding regions (>20 %) across the entire MD trajectory. (C) Binding kinetics measured by isothermal titration calorimetry (ITC) for interactions between 2-3 nm GNP and VKD proteins (FX, FIX and PC). (D) Western blot analysis of GNP-protein complexes formed after incubation of 2-3 nm GNPs with FX-supplemented or FX-deficient plasma, probed for FX. Following plasma incubation and protein separation, multiple washing steps were performed, and the corresponding flow-throughs and retentate were analyzed to determine the FX content. (For interpretation of the references to colour in this figure legend, the reader is referred to the Web version of this article.)

Although negatively charged GNPs can recognize the Gla domains of VKD coagulation proteins, their interaction strength varies considerably (Fig. 1B). GNPs smaller than 2 nm exhibit transient, random interactions around the Gla domain via Ca^2+^ bridging (Fig. S5A and Fig. S9). As GNP size increased (from <2 nm to 2.8 nm), stronger interactions with Gla domains were observed (Fig. 1B and Fig. S4 and S5). Notably, 2.8 nm GNPs bound to both Gla and functional domains of FX and formed a stable locked conformation with FX, whereas their interactions with FIX and PC primarily involved Ca^2+^ coordination within the Gla domain alone (Fig. 3A and B and S5B). Therefore, 2.8 nm GNPs exhibited tighter binding to FX, supported by residue contact number and interaction energy analysis (Fig. 1B and Fig. S4). Larger GNPs (notably 6.1 nm) demonstrated increased off-target interactions (EGF and functional domains), potentially reducing their binding specificity (Fig. S5 C-D and S9). The locked conformation between 2.8 nm GNPs and FX was not observed with other particle sizes, likely due to geometric incompatibility with the Gla domain and adjacent regions. Thus, while negatively charged GNPs in general can interact with the Gla domain, only particles within the 2–3 nm range form a stable and functionally relevant interaction with FX, as supported by fluorescence colocalization (Fig. 2).

Given these observations, we next investigated whether intrinsic structural differences among VKD proteins contribute to their distinct GNP-binding behaviors. While FIX, FX, and PC share similar structural organizations (Fig. S1), 200 ns MD simulations revealed pronounced conformational heterogeneity (Fig. S3), quantified by structural indices (Fig. S10A–B). In addition, surface charge distribution, hydrophobicity, and hydrophilicity varied substantially among these proteins (Fig. S10C–D). Together, these differences explain why GNPs of an optimal size (2–3 nm) form a stable locked conformation with FX, while interactions with other VKD proteins remain weak.

To validate the targeting capacity of 2-3 nm GNPs for FX experimentally, ITC analysis was also used to quantify the binding affinities and thermodynamics of the interactions between the GNPs and VKD coagulation proteins (Fig. 3C). The experimental data and best-fit binding curves for VKD coagulation proteins provided stoichiometry for association constants (Ka), dissociation constants (Kd), enthalpy (ΔH), entropy (ΔS) changes and the number of binding sites (N) (Table S1). Among these, Ka is the most important index for evaluating the binding strength of GNP with VKD coagulation proteins. The Ka for FX is 16.39 × 10^5^ M^−1^, more than four times higher than FIX and PC, indicating a greater binding affinity, as supported by its lower Kd. The ITC results closely match our simulations, suggesting that the interaction between GNPs and VKD coagulation proteins is strongly influenced by structural differences dictated by protein conformational states, with a specific nanoparticle dimension allowing strong and stable binding. The structure-dependent binding is further supported by previous Western blot results (Fig. 1E and F, Fig. S7).

As is well known, the plasma environment is highly complex, containing diverse proteins and other components. Therefore, verifying the specificity of GNP-FX binding under such conditions is essential. Compared to FX-deficient plasma, large-molecular-weight GNP-protein complexes were clearly detected after incubation with FX-supplemented plasma by Western blot using anti-FX antibodies (Fig. 3D). The concentration of FX in the complex increased over time, indicating continuous adsorption of FX onto GNPs (Fig. S11A). Similar complexes were also detected by probing for HSA (Fig. S11A–B), indicating that upon entering plasma, GNPs can bind FX as well as other abundant proteins to form large assemblies. However, the binding strengths differed substantially. After multiple washing cycles, only minimal FX was detected in the washing buffer and showed a declining trend (Fig. 3D and Fig. S11C–D), reflecting a strong and stable association. In contrast, abundant proteins such as HSA remained consistently detectable in the washing buffer after repeated washes, indicating weaker and reversible interactions (Fig. S11C–D), consistent with the findings reported by Cedervall *et al.* [43]. More importantly, FIX within the complexes was readily removed during washing, leaving only trace amounts in the final retentate (Fig. S11 C-D), thereby reinforcing the selective association of 2-3 nm GNPs with FX among vitamin K–dependent coagulation proteins. Collectively, these results confirm that 2–3 nm GNPs preferentially and selectively target FX, even within the complex plasma environment, highlighting their potential for the prevention and treatment of VTE.

### 3.3 Targeted modulation of FX structure and function by 2-3 nm GNPs

**Fig. 4:**
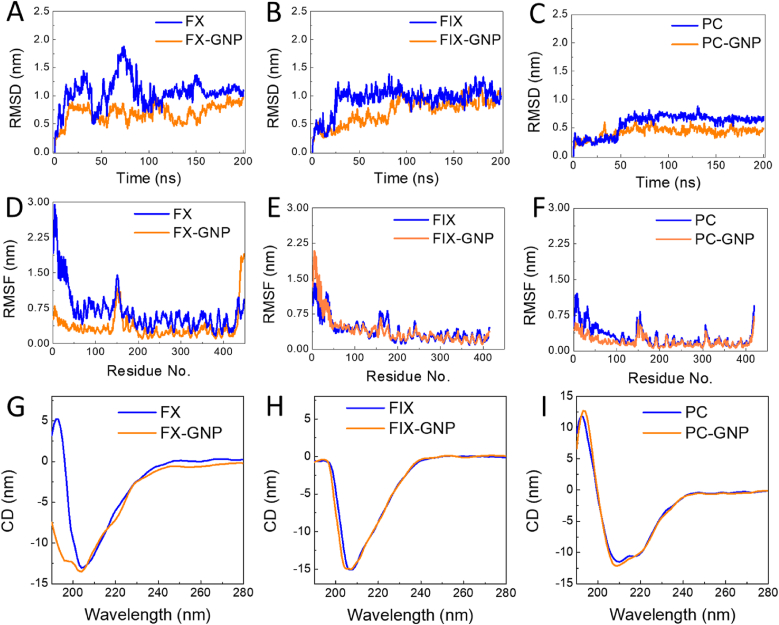
Influence of 2-3 nm GNPs on conformational changes of VKD coagulation proteins. (A–C) RMSD time evolutions during 200 ns MD simulation for VKD coagulation proteins in the presence or absence of 2-3 nm GNPs. (D–F) RMSF values of VKD coagulation proteins calculated based on 200 ns simulations. (G–I) CD spectra of VKD coagulation proteins showing secondary structural change after interacting with or without 2–3 nm GNPs.

Encouraged by the above results, we further investigated the impact of 2-3 nm GNPs binding to FX on its functional and structural properties. Likewise, FIX and PC were also used for comparison. As shown in MD results, GNPs bind to FIX and PC mainly through Ca^2+^ bridge (Fig. 3 A–B, S5 and S9), therefore they do not directly interact with proteins themselves. However, the 2–3 nm GNPs can be wrapped by full FX, resulting in an increased surface contact area (Fig. 3B and Fig. S12) and significant conformational changes (Fig. S13). Due to extensive interactions across multiple FX regions and the formation of a locked conformation, the overall structure of FX was constrained, as evidenced by reduced root mean square displacement (RMSD) (Fig. 4 A) and decreased root mean square fluctuation (RMSF) in most residues (Fig. 4D). Only the local loop in the C-terminal region exhibited increased flexibility, reflecting its greater conformational freedom, without affecting the overall conformational constraint of FX. Cross-correlation between FX's EGF and functional domains was also significantly enhanced by GNP binding (Fig. S14). These structural changes in FX upon GNPs interaction were experimentally confirmed using CD spectroscopy (Fig. 4G) and supported by secondary structure changes in MD simulation (Fig. S15 A-B). In contrast, FIX and PC retained greater structural freedom post-binding, exhibiting minimal conformational changes (Fig. 4 and Figs. S14–S15).

**Fig. 5:**
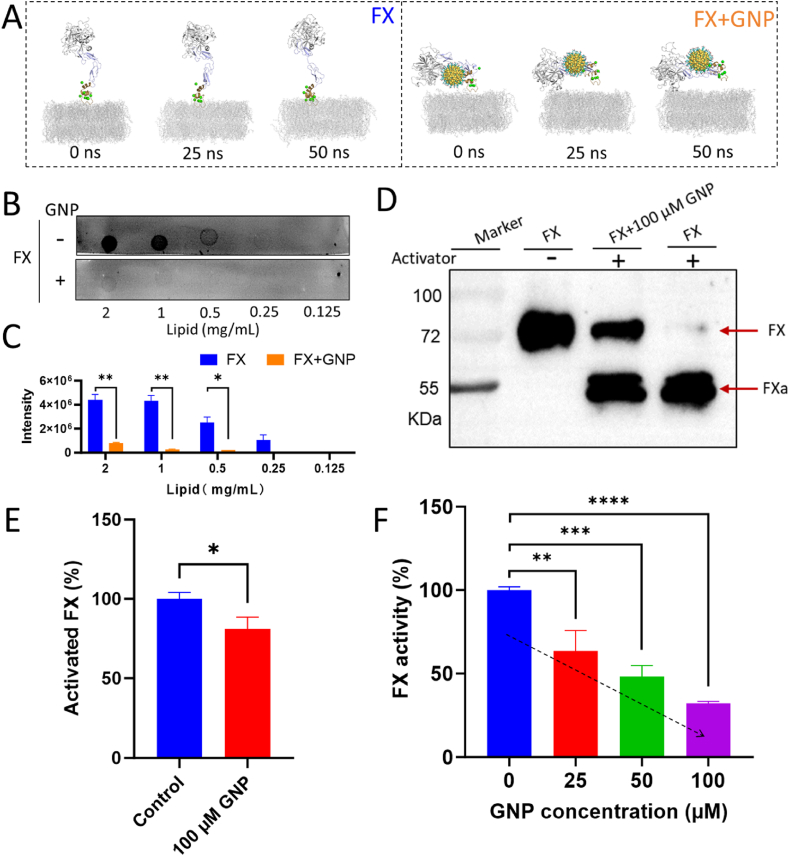
Functional impact of 2-3 nm GNP on FX. (A) Time evolutions of typical simulation snapshots with or without 2.8 nm GNP. (B) Protein-lipid overlay assay evaluating FX's ability to bind the lipid membrane in the absence and presence of GNPs. (C) Quantitative analysis of FX binding to liposomes, based on grey values. (D) Western blot results of FX activation with or without 100 μM GNPs. (E) Quantitative analysis (ImageJ-based) of FX cleavage affected by 2-3 nm GNPs, based on grey values in (D). (F) Concentration-dependent inhibition of FX enzymatic activity by varying concentrations (0–100 μM) of GNPs using a chromogenic substrate method. ∗*p* < 0.05, ∗∗*p* < 0.01, ∗∗∗*p* < 0.001, ∗∗∗∗*p* < 0.0001.

Direct oral anticoagulants (DOACs) inhibit FX by binding to the active site within its serine protease domain, representing the conventional therapeutic strategy. To assess potential mechanistic overlap, we conducted molecular simulations comparing the rivaroxaban-binding pocket (Fig. S16A–C) with the GNP-binding region in FX (Fig. 3B). The two sites were found to be spatially distinct, suggesting minimal competition between DOACs and GNPs for FX binding (Fig. S16D). In conjunction with the conformational constraints previously observed upon GNP association, these findings indicate that 2–3 nm GNPs impair FX function predominantly by restricting its global conformational dynamics rather than by direct active-site inhibition. As we know, the Gla domain's interaction with lipid membranes is critical for FX function in the coagulation cascade [53]. We hypothesized that dynamics restriction imposed by GNP binding might impair FX's lipid-binding efficiency. Additional MD simulations examining FX interactions with lipid membranes, both free and complexed with GNP, supported this hypothesis. As shown in Fig. 5A, the Gla domain of free FX binds to the lipid membrane, positioning FX upright on the membrane, where it interacts with other coagulation factors to perform its function [54]. However, FX bound to GNP exhibited significantly diminished membrane interactions (Fig. S17A), preventing the Gla domain of FX from attaching to the membrane surface (Fig. 5A and Fig. S18). Therefore, the tilt angle ψ of FX changed markedly from ∼0° to ∼90° (Fig. S18A), and the centroid of the functional domain to the lipid membrane surface reducing from 9.5 nm to 3.5 nm (Fig. S18B), substantially impairing FX's ability to participate in coagulation complexes. These computational findings were experimentally validated by protein-lipid overlay (PLO) assay [45]. As shown in Fig. 5B, FX binds to liposomes in the absence of GNPs, but fails to bind when GNPs are present. Quantitative analysis confirms that GNPs can significantly inhibit FX binding to lipid membranes (Fig. 5C).

Given that GNP binding involved FX's functional domains alongside its Gla domain, we speculated that FX's enzymatic activity and activation efficiency might also be affected. Western blot assays confirmed significant inhibition of FX activation upon exposure to GNPs, with activated FX (FXa) levels markedly reduced at 100 μM GNP (Fig. 5D and E). In contrast, larger GNPs (>8 nm) showed no apparent interference with FX activation (Fig. S19A–B). Consistently, enzymatic activity assays revealed a concentration-dependent suppression of FX activity, with approximately 60 % inhibition at 100 μM 2–3 nm GNP (Fig. 5F), whereas larger GNPs exhibited no detectable inhibitory effect (Fig. S19C). Additionally, at 100 μM GNP, measurable effects on both FIXa and thrombin activity were not observed (Fig. S20). These results collectively demonstrate a size-dependent nature of this functionally relevant interaction and highlight the effectiveness of 2-3 nm GNPs in impeding FX's pivotal role in the blood coagulation cascade.

### 3.4 Potential of 2-3 nm GNPs in treatment and prevention of VTE

**Fig. 6:**
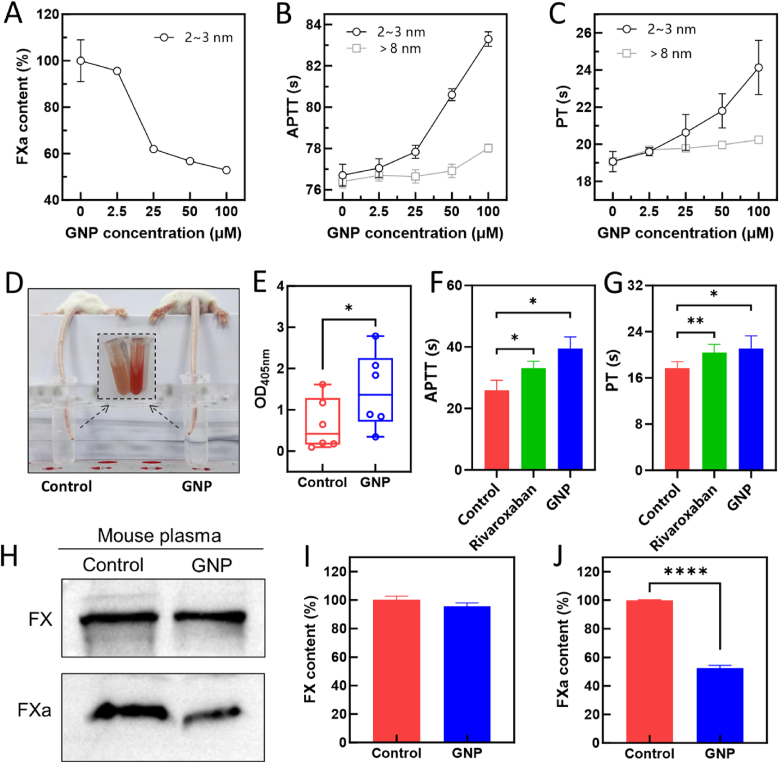
*In vitro* and *in vivo* anticoagulant activity of 2-3 nm GNPs targeting FX. (A) ELISA-based quantification (FXa ELISA kit) of FXa levels in standard human plasma samples incubated with varying concentrations of GNPs. (B–C) Effect of GNPs (2–3 nm and >8 nm) on plasma clotting times, assessed by APTT (B) and PT (C) assays in standard human plasma. (D) Representative image from mouse tail-bleeding experiments, with inset illustrating accumulated blood volume post-tail cutting. (E) Quantitative analysis of bleeding ( OD_405nm_) in mice (n = 6/group) 3 h after injection with or without GNPs. Data are presented means ± SD, n = 6. (F–G) Quantification of clotting times by APTT (F) and PT (G) assays in plasma from mice treated for 7 days with GNPs (daily intravenous injection, 100 μL of 100 μM), rivaroxaban (daily oral administration, 300 μL of 0.5 mg/mL), or normal saline (daily intravenous injection, 100 μL). (H) Western blot analysis of total FX and FXa in mouse plasma from tail-bleeding assay using Factor X Rabbit mAb and Cleaved-F10 (A41) antibody, respectively. (I–J) Quantitative analysis of taotal FX (I) and FXa (J) from Western blot bands (panel H), quantified via ImageJ software. ∗*p* < 0.05, ∗∗∗∗*p* < 0.0001.

The experimental and simulation findings demonstrate that 2–3 nm negatively charged GNPs targeting FX could significantly suppress both FX activation and enzymatic activity, as well as impair its membrane-binding capacity, suggesting potential as an effective anticoagulant. To further validate this therapeutic potential, we examined their effect on FXa levels in standardized human plasma using ELISA assay (Fig. 6A). The corresponding coagulation times, including APTT and PT, are presented in Fig. 6B and C. At GNP concentrations below 25 μM, sufficient free FXa remains available to sustain the coagulation cascade, resulting in only a moderate prolongation of both APTT (Fig. 6B) and PT (Fig. 6C). As the GNP concentration increased from 25 μM to 100 μM, a progressively larger fraction of FX was bound or sequestered by the particles, thereby reducing the pool of functionally available FXa. Although single-point chromogenic assays indicated only a slight reduction in FXa activity, its effective contribution to the cascade was compromised, leading to a pronounced prolongation of clotting times, particularly evident in the APTT assay (Fig. 6B). In contrast, larger GNPs (>8 nm) induced negligible changes in either APTT or PT, further underscoring the size-dependent nature of this functionally relevant interaction (Fig. 6B and C).

To verify antithrombotic effects *in vivo*, a tail‐bleeding assay was performed in the mouse model [55] (Fig. 6D). Twelve BALB/c mice were divided evenly into experimental and control groups (6 each), receiving intravenous injections of either GNP (100 μM) or normal saline (control). Tail bleeding volume significantly increased (p < 0.05) in the GNP-treated group 3 h after treatment, as quantified by OD_405nm_ measurements (Fig. 6E), indicating effective anticoagulation. Plasma samples collected post-treatment further confirmed significant prolongation of APTT and PT compared to controls (Fig. S21 A-B). Clotting times were further compared with the positive control rivaroxaban after one week of treatment. Both APTT and PT assays demonstrated significant prolongation with rivaroxaban (daily oral administration, 300 μL of 0.5 mg/mL); however, the effect was weaker than that induced by 2-3 nm GNPs (daily intravenous injection, 100 μL of 100 μM) (Fig. 6F and G). Western blot analysis showed similar total FX levels but significantly decreased FXa (48 % reduction) in the GNP-treated group (Fig. 6H–J), exhibiting selective inhibition of FX activation.

**Fig. 7:**
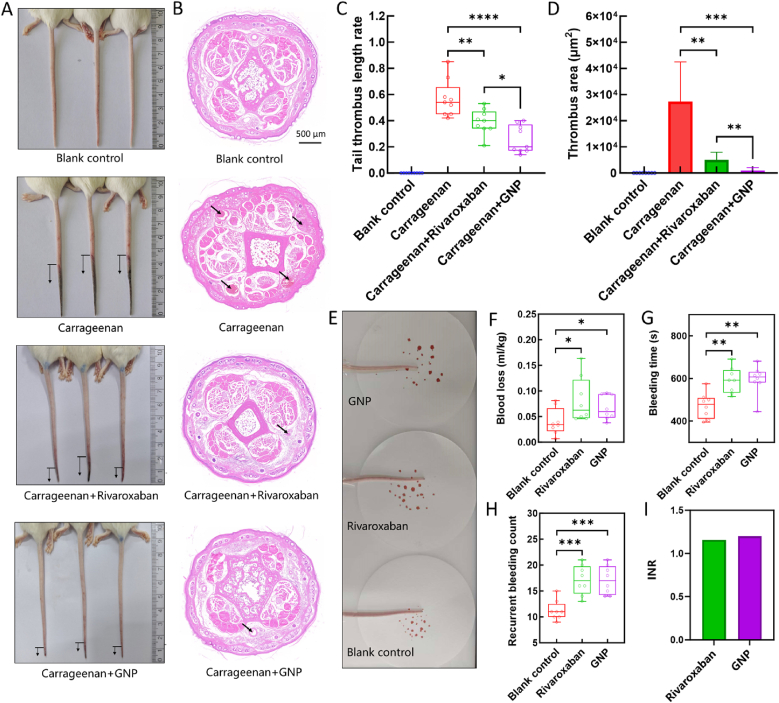
Therapeutic efficacy of 2-3 nm GNPs in a mouse model of venous thrombosis. (A) Representative photographic images of mouse tails following carrageenan-induced thrombosis, comparing GNP-, rivaroxaban- and normal saline-treated groups. (B) Representative histopathological (H&E-stained) images of mouse tail sections, with arrows indicating thrombus formation in each group. (C) Statistical comparison (mean ± SD, n = 9/group) of thrombus length (% tail length) between groups, demonstrating antithrombotic efficacy of GNP treatment. (D) Thrombus area was quantitatively assessed by area-based analysis of H&E-stained sections (nine images per group). (E) Representative photographs illustrating the hemostatic effects of GNP, rivaroxaban and normal saline in the rat tail amputation model. (F–H) Quantitative analysis of blood loss (F), bleeding time (G), and recurrent bleeding count (H) in rats treated with GNP, rivaroxaban and normal saline. Data are presented means ± SD, n = 8/group. (I) Calculated INR values for rivaroxaban and GNPs. ∗*p* < 0.05, ∗∗*p* < 0.01, ∗∗∗*p* < 0.001, ∗∗∗∗*p* < 0.0001.

Encouraged by these anticoagulant effects, we conducted a carrageenan-induced mouse thrombus model to investigate the antithrombotic activity of 2-3 nm GNPs *in vivo* [49] (Fig. S22). Mice (n = 36) were administered either daily intravenous injections of GNPs (100 μM, 100 μL), daily oral rivaroxaban (0.5 mg/mL, 300 μL), or daily intravenous injections of normal saline (100 μL). As shown in Fig. 7A–C, thrombosis formation was significantly attenuated in the GNP-treated group (26 % tail-length thrombus occupancy) compared to blank control (57 % occupancy) and rivaroxaban (39 % occupancy). Histological (H&E) analysis further confirmed reduced thrombus formation and significantly decreased thrombus area in GNP-treated tails (Fig. 7 B and D).

Following confirmation of the potent *in vivo* antithrombotic activity of 2-3 nm GNPs, we next assessed bleeding safety using a mouse tail-bleeding assay (Fig. 7E). All parameters—including blood loss (Fig. 7F), bleeding time (Fig. 7G), and recurrent bleeding events (Fig. 7H)—were elevated in both rivaroxaban- and GNP-treated groups compared with controls. Importantly, the magnitude of these increases with 2-3 nm GNPs was comparable to rivaroxaban, a clinically approved anticoagulant, indicating no additional bleeding risk. This conclusion is further supported by the lower INR observed in the GNP group, which was similar to that of rivaroxaban (Fig. 7I).

Finally, we evaluated the biocompatibility of 2-3 nm GNPs at both cellular and organismal levels. *In vitro* cytotoxicity assays in HEK293 and HepG2 cells showed no significant toxicity at concentrations up to 200 μM (Fig. S23A–B). *In vivo* biodistribution analysis further revealed consistently low Au accumulation in the brain (Fig. S24A), indicating that only small amounts of GNPs crossed the blood-brain barrier [56]. The Au content in the blood gradually decreased over time, while levels increased in the liver without significant accumulation in the kidney. These findings are consistent with the widely reported observation that GNPs mainly accumulate in the liver and are renal clearance [57,58]. Given that many coagulation factors are synthesized in the liver, the hepatic accumulation of GNPs suggests a degree of functional targeting for VKD coagulation proteins [59]. Additionally, we monitored the Au content in the mice feces following both a single injection and continuous injections. After a single injection, the Au content in feces decreased over time, reflecting systemic clearance (Fig. S24B). Notably, repeated injections resulted in sustained Au levels, suggesting continuous metabolic excretion (Fig. S24C). Furthermore, histopathological examination of kidney and liver tissues (Fig. S24D–E) revealed no evidence of tissue damage or inflammatory lesions in the GNP-treated group, with morphology comparable to both saline- and rivaroxaban-treated controls. This result is consistent with previous reports indicating that ultrasmall, negatively charged AuNPs typically elicit minimal immune stimulation *in vivo* [[60], [61], [62]]. Consistently, liver enzyme levels remained unaffected, as indicated by comparable AST (Fig. S24F) and ALT (Fig. S24G) concentrations across all treatment groups. Overall, 2–3 nm GNPs exhibit excellent biocompatibility, positioning them as a promising antithrombotic therapeutic platform.

## 4 Discussions

Venous thrombosis represents a significant contributor to morbidity and mortality, particularly among the elderly population [4]. VKD coagulation proteins, including coagulation factors II, VII, IX, and X, as well as anticoagulation factors PC, PS, and PZ, are vital for coagulation homeostasis [11]. Targeting specific VKD coagulation proteins allows modulation of the coagulation-anticoagulation balance, offering therapeutic potential in VTE management. Here, we engineered GNPs to selectively regulate coagulation homeostasis by specifically recognizing the VKD coagulation proteins’ Gla domain (Fig. 1).

VKD proteins contain a specialized domain rich in glutamic acid residues, catalyzed by γ-glutamyl carboxylase (GGCX) into γ-carboxyglutamic acid (Gla) residues. This modification facilitates the binding of Ca^2+^ to VKD coagulation proteins, inducing conformational changes essential for their biological activity [63,64]. By binding to the Gla domain of VKD coagulation proteins, it is possible to effectively interfere with their conformation, influencing their functional activity [65]. For example, many plasma lipids, such as sphingosine and sphinganine, can bind to the Gla domains of VKD coagulation proteins, disrupting their function and potentially contributing to coagulation disorders [66]. Additionally, antibodies against FIX's Gla domain can disturb its coagulation function, highlighting their antithrombotic therapeutic potential [67].

Numerous studies reported that GNPs’ surface properties critically influence interactions with the coagulation system [24,68]. For instance, RGD (Arginine-Glycine-Aspartic acid)-modified GNPs significantly reduce clotting time when compared to other surface modifications, such as polyethylene glycol （PEG）, human fibrinogen （HFIB）, and clopidogrel （Clop） [69]. Similarly, differences in surface coatings (e.g., citrate, polyallylamine hydrochloride （PAH）, cysteamine, dihydrolipoic acid （DHLA）, PEG) affect binding affinity to albumin and fibrinogen [70]. Additionally, negatively charged polyphosphates released upon platelet activation enhance coagulation by activating the contact pathway of blood clotting and accelerating factor V activation [71,72]. Inspired by these findings, we engineered negatively charged GNPs by introducing mercaptobutyric acid onto their surface, which can specifically recognize and bind to the Gla domain of VKD coagulation proteins (Fig. 1).

Notably, the procoagulant effect of polyphosphates significantly varies depending on the length of the polyphosphate chain [73,74]. In line with this variability, we found that 2–3 nm GNPs specifically target FX while showing weaker binding to other VKD coagulation proteins (Fig. 2). Consistent with a previous report [28], this suggests that the size of nanoparticles can differentially modulate the enzymatic activity of coagulation factor XII, either inhibiting or promoting its activity. GNPs modified with sulfonated chitosan (13 nm) [75], or pyrimidine (10 nm) [51] show prolonged clotting times, inhibit platelet aggregation, and interfere with thrombin and fibrin, suggesting anti-thrombogenic properties. In our study, ultrasmall GNPs, particularly those measuring 2–3 nm, selectively bound and modulated FX, inhibiting coagulation (Fig. 2, Fig. 3, Fig. 4) and effectively blocking the coagulation cascade (Fig. 5, Fig. 6). These results parallel Lira *et al.*’s findings [76], where anionic ultrasmall GNPs exhibit anticoagulant activity by interfering with thrombin-mediated fibrinogen cleavage, though they simultaneously activated Factor XII. We speculate that this apparent discrepancy may be attributed to differences in the surface modifications of the GNPs used in their study compared to those employed in our research. Additionally, another study [29] employing chiral modifications on gold nanoclusters demonstrated differential binding to coagulation factor XII, further supporting our hypothesis.

Precise regulation of coagulation homeostasis is critical for preventing thrombotic events, and targeting VKD coagulation proteins represents a promising strategy. Our results demonstrate that negatively charged GNPs of specific sizes (2–3 nm) can impair FX's enzymatic activity by inducing conformational changes (Fig. 4). Unlike newer oral anticoagulants such as rivaroxaban and apixaban, which exert anticoagulant effects primarily by selectively inhibiting the serine protease activity of FXa [17,77], these nanoparticles offer an alternative mechanism by directly targeting and modifying FX structural conformation. Despite their favorable safety profiles, direct FXa inhibitors may occasionally induce off-target interactions with other serine proteases, potentially leading to rare but severe complications, including gastrointestinal bleeding or pulmonary embolism [19,20]. In contrast, our nanoparticle-based approach highly allows for selective modulation of FX, which was clearly validated by *in vivo* murine experiments demonstrating significant prolongation of clotting times (Fig. 6) and effective antithrombotic outcomes in a venous thrombosis model (Fig. 7). These findings suggest that 2–3 nm GNPs may represent an innovative alternative for the treatment of VTE.

## 5 Conclusion

In summary, we propose a novel antithrombotic strategy employing negatively charged GNPs to selectively regulate the coagulation cascade through selectively interacting with VKD coagulation proteins’ Gla domain. Integrating theoretical calculations with experimental validation, we demonstrate that negatively charged GNPs can recognize the Gla domain of VKD coagulation proteins. Specially, 2–3 nm GNPs exhibit a strong binding affinity to FX, even within the complex plasma environment, effectively suppressing its enzymatic activity, activation efficiency and lipid membrane-binding capacity. *In vivo* studies confirmed that these GNPs significantly attenuate thrombus formation in mouse models, underscoring their potent antithrombotic efficacy. This work offers a novel approach for the prevention and treatment of venous thrombosis.

## CRediT authorship contribution statement

**Shixin Li:** Writing – original draft, Project administration, Methodology, Investigation, Formal analysis, Data curation, Conceptualization. **Yuye Yin:** Writing – original draft, Methodology, Investigation. **Dongmei Hou:** Investigation, Data curation. **Yongchao Jin:** Software, Investigation. **Yuan Zhao:** Investigation, Formal analysis. **Jiangbo Tong:** Software, Investigation. **Xu Liu:** Methodology, Investigation. **Guomin Shen:** Writing – review & editing. **Tongtao Yue:** Writing – review & editing. **Kang Liu:** Investigation. **Yi Gu:** Investigation. **Luju Chen:** Investigation. **Fangzhe Ren:** Investigation. **Jinlin Huang:** Writing – review & editing, Resources, Project administration, Methodology. **Jian-Ke Tie:** Writing – review & editing, Resources, Project administration, Methodology. **Zhenyu Hao:** Writing – review & editing, Writing – original draft, Validation, Resources, Project administration, Methodology.

## Declaration of competing interest

The authors declare that they have no known competing financial interests or personal relationships that could have appeared to influence the work reported in this paper.

## Data Availability

Data will be made available on request.
