# Supplementary material for: Selective targeting of coagulation factor X Gla domain by negatively charged gold nanoparticles: a novel method for controlled antithrombotic therapy
Source: Mater Today Bio. 2025 Oct 1;35:102378. doi: 10.1016/j.mtbio.2025.102378 (PMC12528873; doi:10.1016/j.mtbio.2025.102378)
Supplement: Multimedia component 1 [file mmc1.docx]

**Supplemental Materials**

**Selective targeting of coagulation factor X Gla domain by negatively charged gold nanoparticles: a novel method for controlled antithrombotic therapy**

Shixin Li ^a,1^, Yuye Yin ^a,1^, Dongmei Hou ^a^, Yongchao Jin ^a^, Yuan Zhao ^a^, Jiangbo Tong ^a^, Xu Liu ^b^, Guomin Shen ^c^, Tongtao Yue ^d,e^, Kang Liu ^a^, Yi Gu ^a^, Luju Chen ^a^, Fangzhe Ren ^a,f^, Jinlin Huang ^a,f,*^, Jian-Ke Tie ^g,**^, Zhenyu Hao ^a,h,***^

*^a^ College of Bioscience and Biotechnology, Yangzhou University, Yangzhou, 225009, China*

*^b^ College of Chemistry and Chemical Engineering, Yangzhou University, Yangzhou, 225009, China*

*^c^ Department of Cell Biology, School of Basic Medical Sciences, Harbin Medical University, Harbin, 150081, China*

*^d^ Institute of Coastal Environmental Pollution Control, Key Laboratory of Marine Environment and Ecology, Ministry of Education, Ocean University of China, Qingdao, Shandong, 266100, China*

*^e^ Laboratory for Marine Ecology and Environmental Science, Qingdao National Laboratory for Marine Science and Technology, Qingdao, Shandong, 266237, China*

*^f^ Joint International Research Laboratory of Agriculture and Agri-Product Safety, Ministry of Education of China, Yangzhou, Jiangsu, 225009, China*

*^g^ Department of Biology, The University of North Carolina at Chapel Hill, Chapel Hill, NC, 27599, USA*

*^h^ Affiliated Hospital, Yangzhou University, Yangzhou, Jiangsu 225009, China*

^*^**Corresponding author.** College of Bioscience and Biotechnology, Yangzhou University, Yangzhou, 225009, China.

^**^**Corresponding author.**

^***^**Corresponding author.** College of Bioscience and Biotechnology, Yangzhou University, Yangzhou, 225009, China.

*E-mail addresses*: jinlin@yzu.edu.cn (J. Huang), jktie@email.unc.edu (J.-K. Tie), zhyuhao@hotmail.com (Z. Hao)

^1^ These authors contributed equally to this work.

**The** **Supplemental Data includes:**

Supplemental Fig. S1-S24, Supplemental Table 1.

**Methods**

**Molecular docking simulations**

The initial models of vitamin K dependent (VKD) coagulation proteins, including FIX, FX, and PC, were generated with AlphaFold2 [1]. Each configuration was equilibrated for 100 ns, following Ca^2+^ addition to the Gla domain. The stable structures were then used for molecular docking simulations. GNP models were made with CHARMM-GUI [2]. The VKD coagulation proteins and GNP models were processed with AutoDockTools to prepare input files for docking simulation [3]. Polar hydrogens were added to the receptor protein, and the Kollman-United atom charges were employed. All molecular structures were saved in PDBQT format for molecular docking simulations. Molecular docking simulations were conducted with AutoDock Vina [4] (version 1.1.2). The GNP docking search space covered the entire protein surface using a grid box of 12.6 × 12.6 × 12.6 nm³. Ten binding conformations were provided for each GNP-protein complex based on docking affinity. The screened complexes were analyzed for binding energy and poses, then subjected to molecular dynamics simulations for further valuation.

**Molecular dynamics simulations**

All simulations were conducted using GROMACS 2019-3 [5]. The initial structure of rivaroxaban-FXa for molecular dynamics simulation was based on the crystal structure (PDB ID: 2W26). The rivaroxaban-FXa or GNP-protein complex was initially centered in a cubic box, over 1.5 nm distance from the boundary. The complexes were solvated in TIP3P water and neutralized with NaCl. The CHARMM field, generated through CHARMM-GUI [2], was used for our simulations. The system was energy-minimized using the steepest descent method until convergence. The system was then equilibrated in the NVT ensemble (constant bead number, volume, and temperature) for 1 ns. Subsequently, the system was equilibrated under the NPT ensemble (constant bead number, pressure, and temperature). Pressure was held at 1 bar using the isotropic or simiisotropic Parrinello-Rahman barostat, and temperature was maintained at 310 K with a Nose-Hoover thermostat, and coupling constant is 4 ps. Short-range interactions were defined with a 1.2 nm cutoff, using a Lennard-Jones potential smoothly shifted to zero between 0.9 nm and 1.2 nm to reduce cutoff noise. Long-range electrostatic interactions were managed with the particle-mesh Ewald summation method. Periodic boundary conditions were applied in all dimensions. The time step was 10 fs, and the neighbor list was updated every ten steps. Visualizations were created with PyMOL [6].

**Cell toxicity assay**

The cytotoxicity of GNPs was evaluated in HEK293 and HepG2 cells over a concentration range of 0-200 μM. Cells were incubated with GNPs for 24 h, and viability was assessed using a standard CCK-8 assay [7].


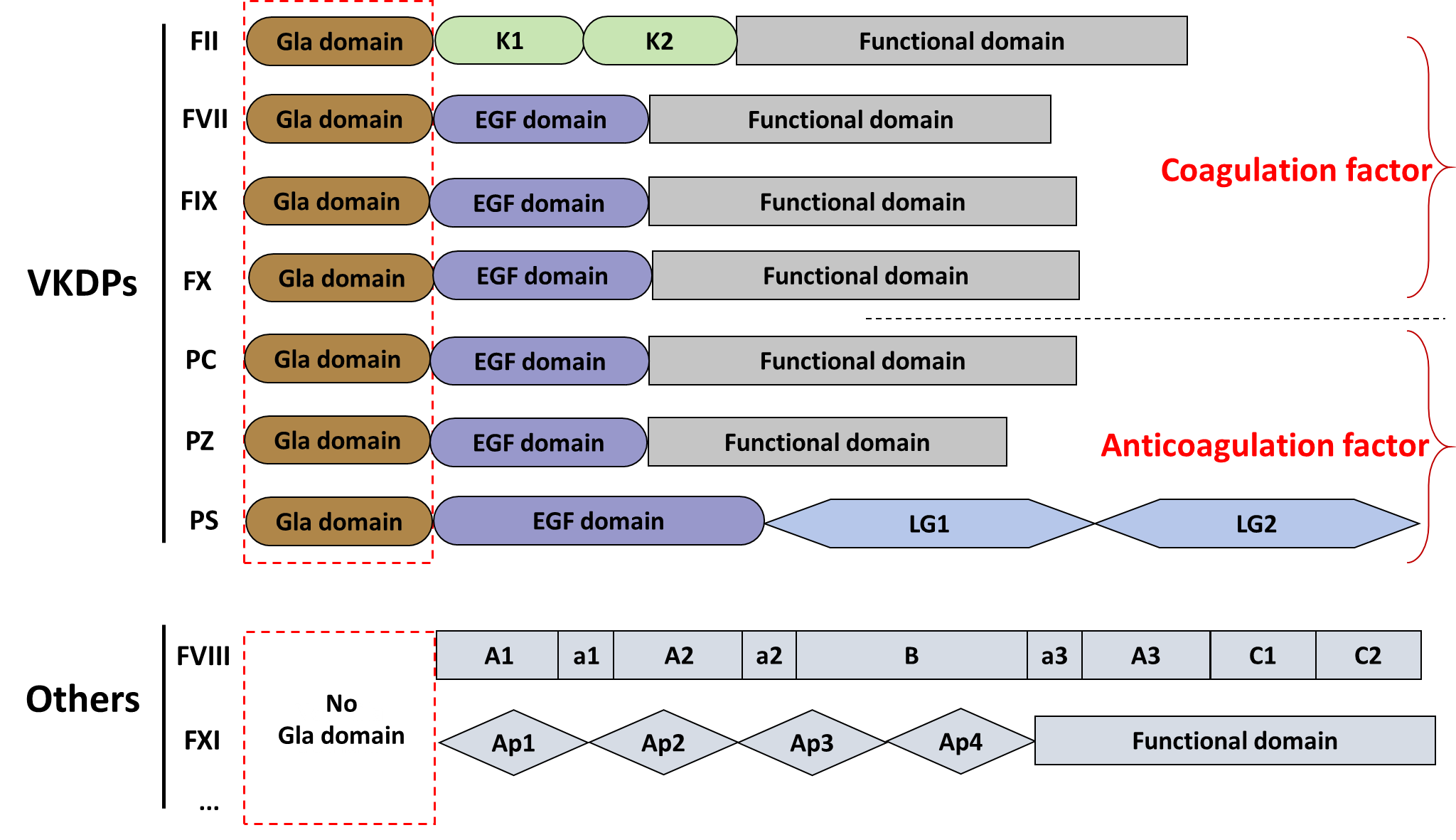


**Fig. S1.** Structural domains of various VKD coagulation proteins and other proteins.


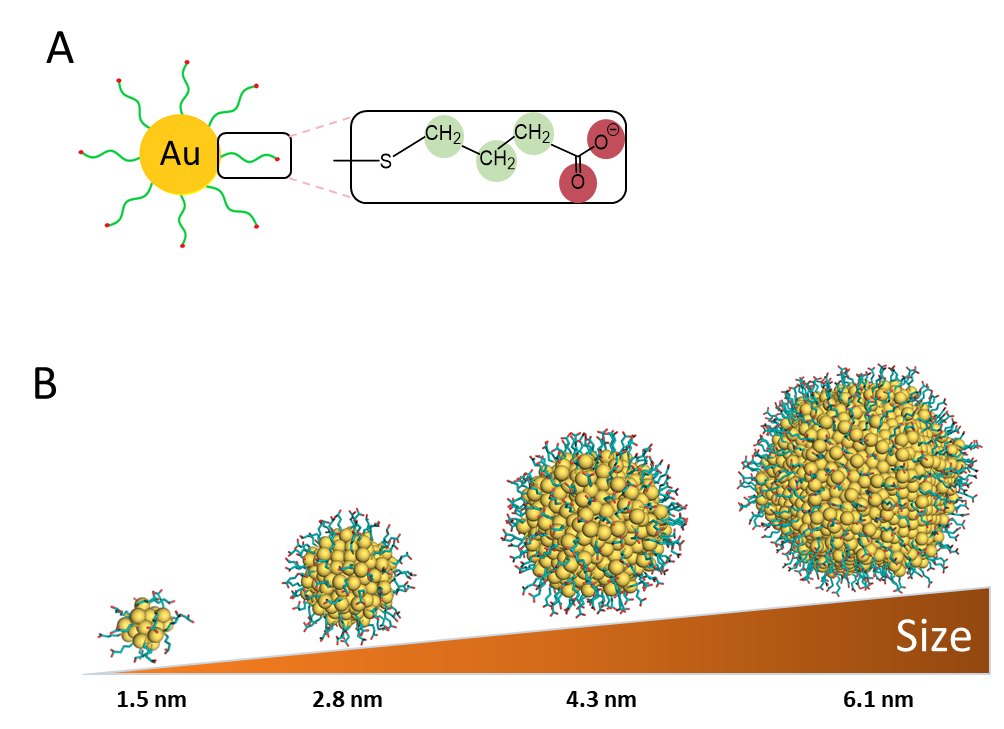


**Fig. S2.** (A) Schematic representation of molecular structure of GNPs. (B) GNPs of various sizes used in our simulations.


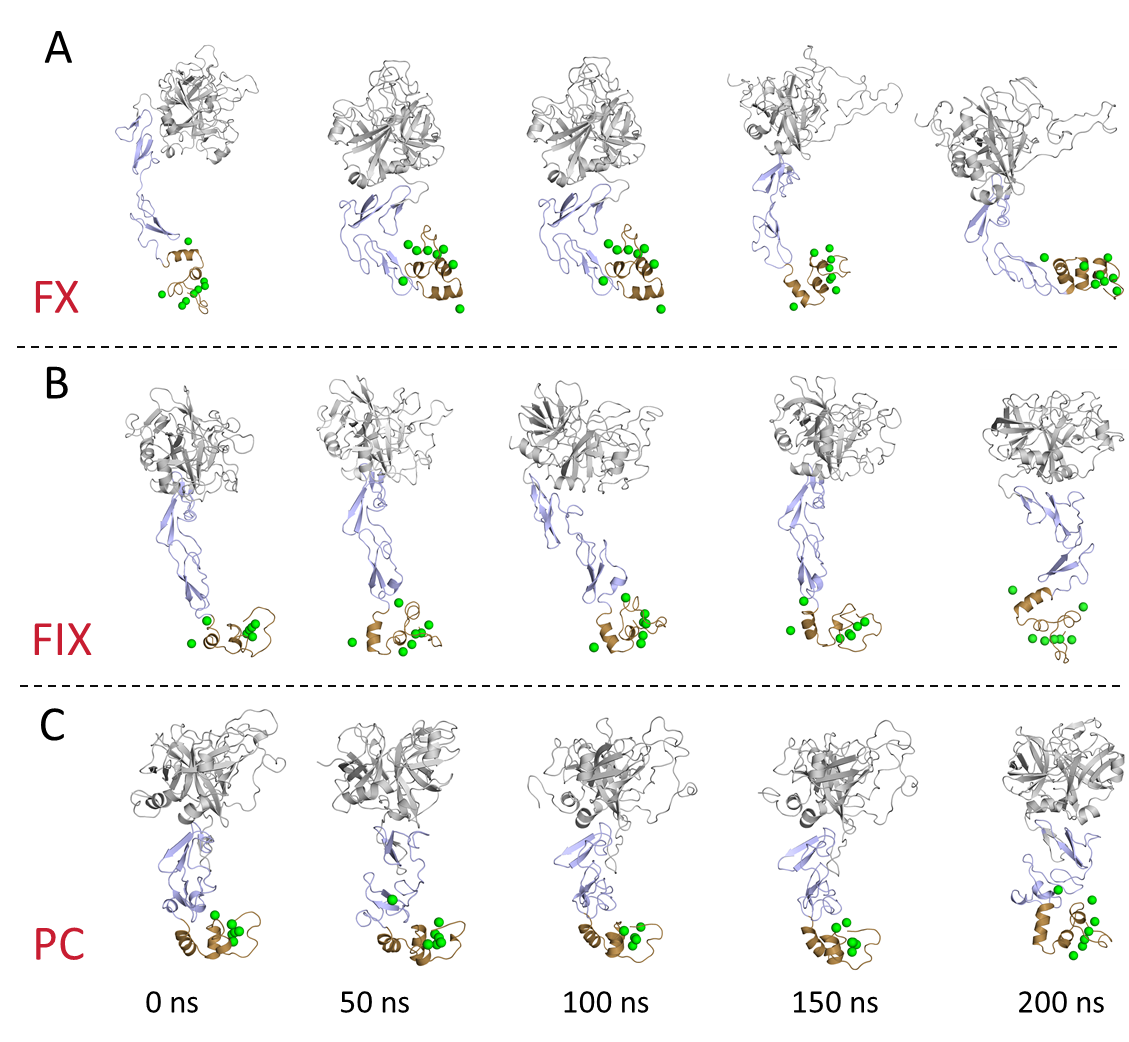


**Fig. S3.** Time evolutions of typical snapshots of VKD coagulation proteins moving freely in water. (A) Factor X (FX), (B) factor IX (FIX), (C) protein C (PC).


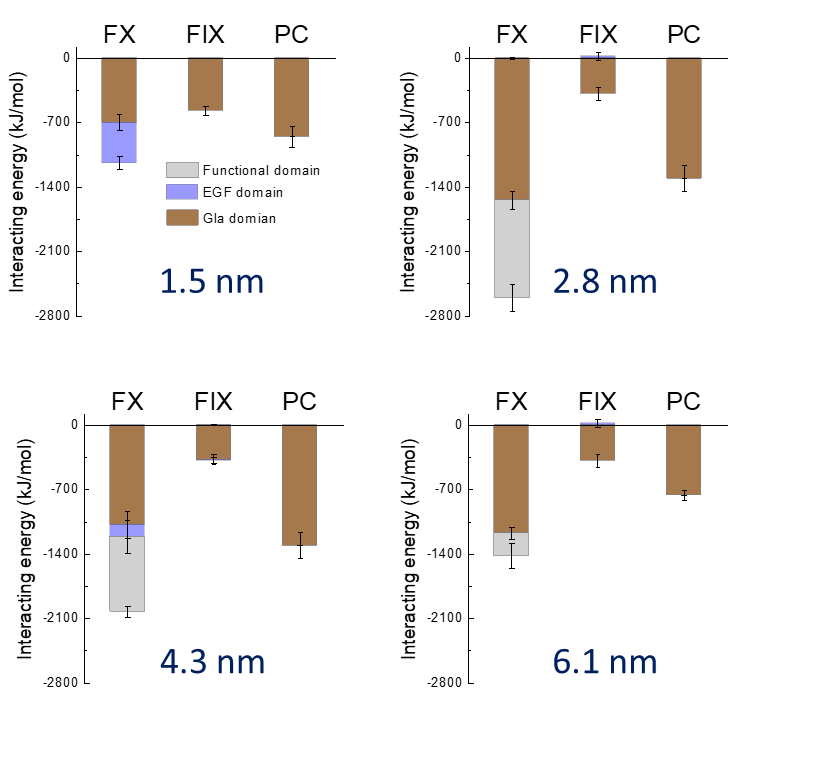


**Fig. S4.** Interacting energy contributions of VKD coagulation protein’s structural domains with GNPs of various sizes.


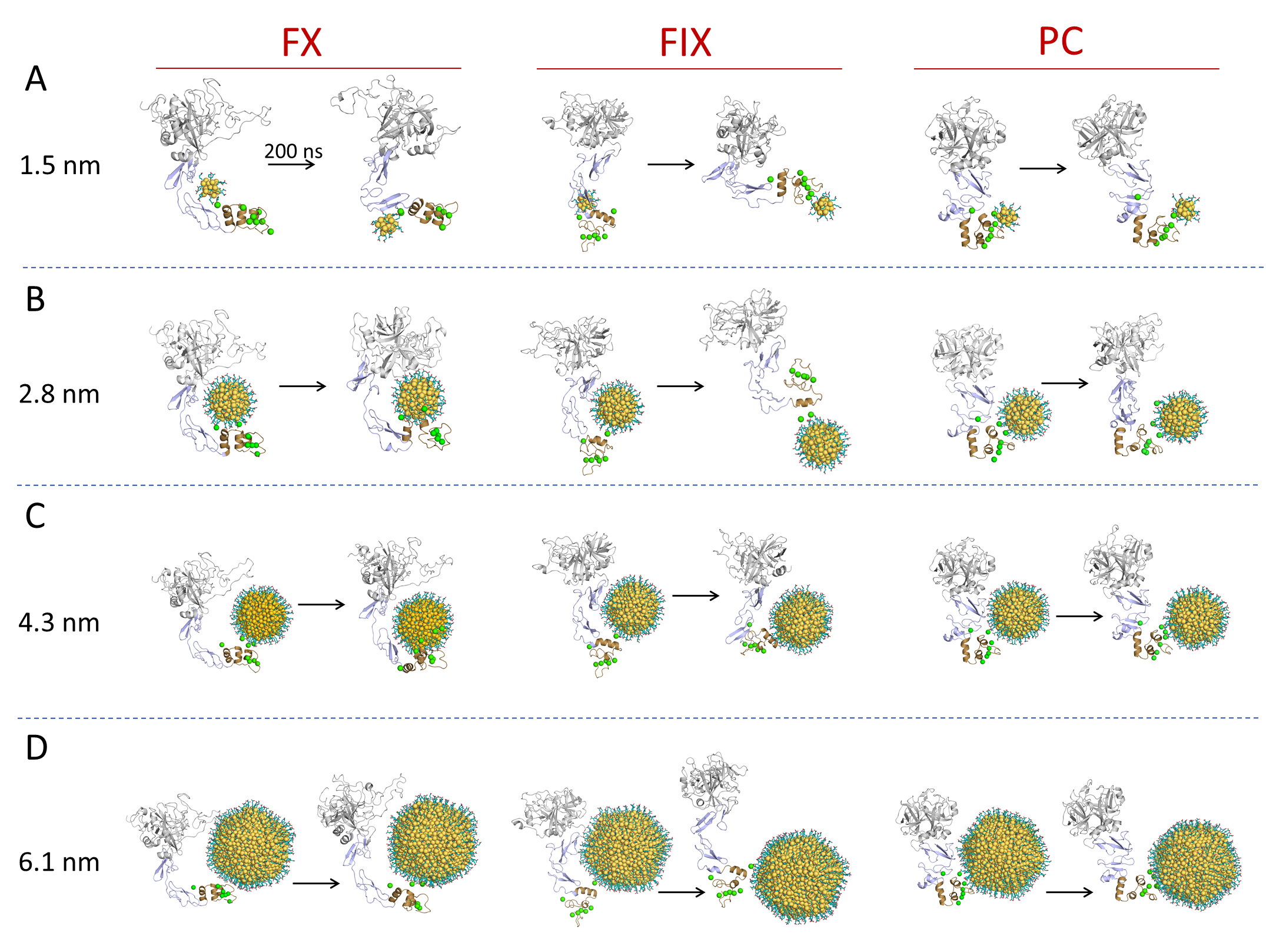


**Fig. S5.** Initial and final snapshots of binding conformations of VKD coagulation proteins with various GNPs during 200 ns MD simulations. The initial structures were obtained from molecular docking simulations.


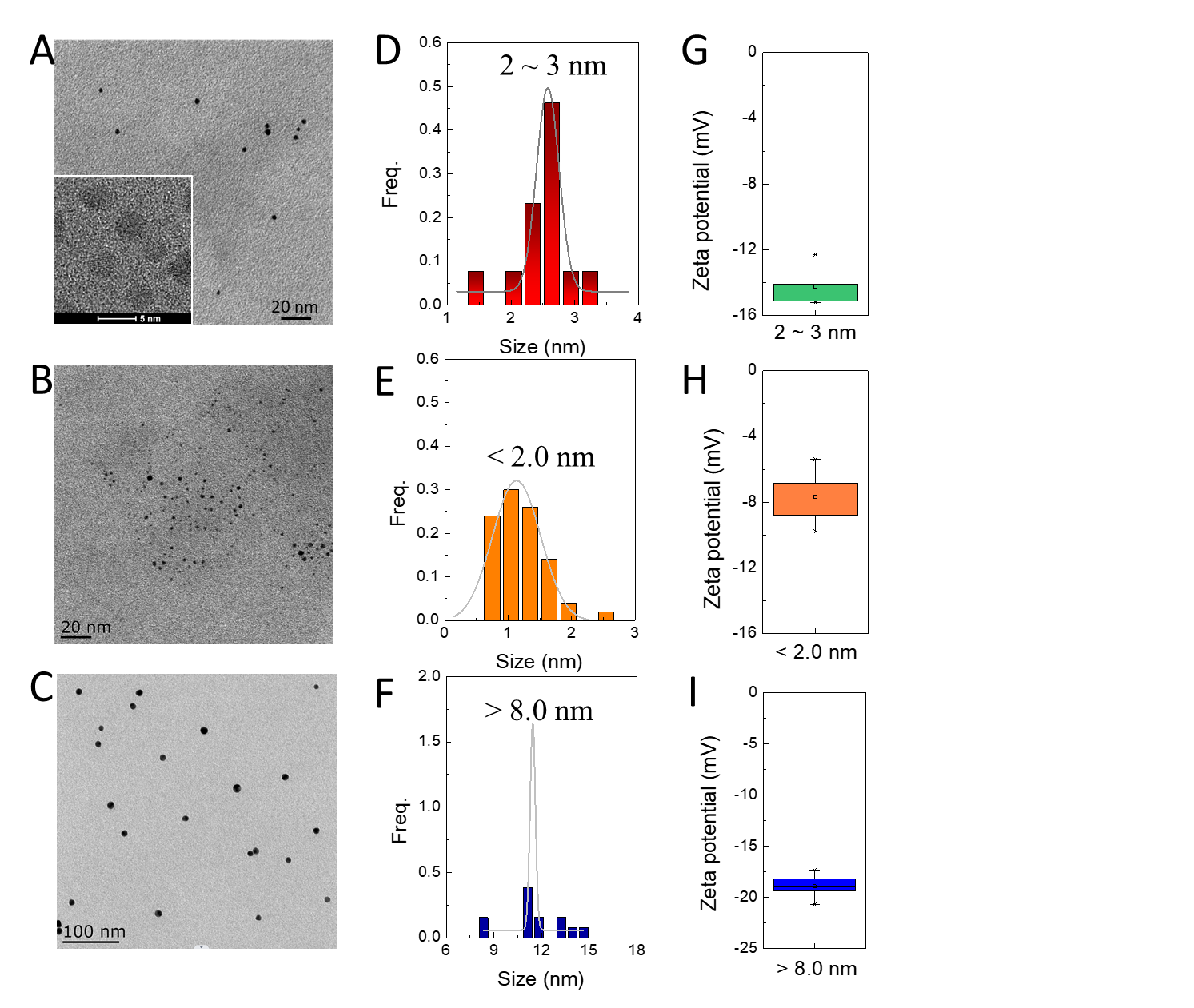


**Fig. S6.** Synthesis and characterization of GNPs of various sizes. (A-C) TEM characterization, (D-F) size distribution, and (G-I) zeta potential.


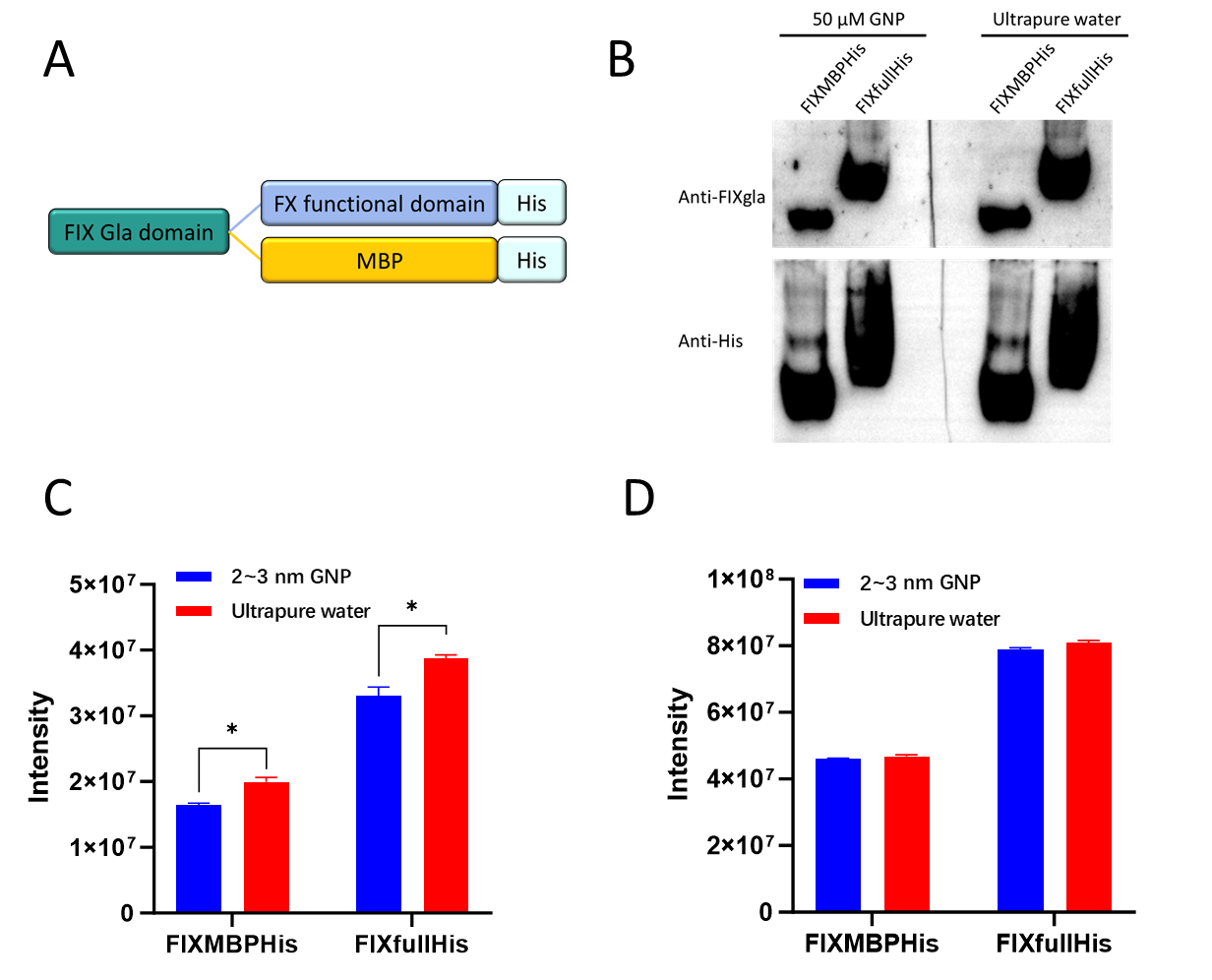


**Fig. S7.** (A) Topology diagram of distinct reporter proteins. (B) Western blot results showing the binding differences of 2-3 nm GNPs with various VKD coagulation proteins. (C-D) Quantitative analysis of FIX content, based on grey values, detected using Anti-FIXgla antibody (C) or Anti-His antibody (D). **p* < 0.05.


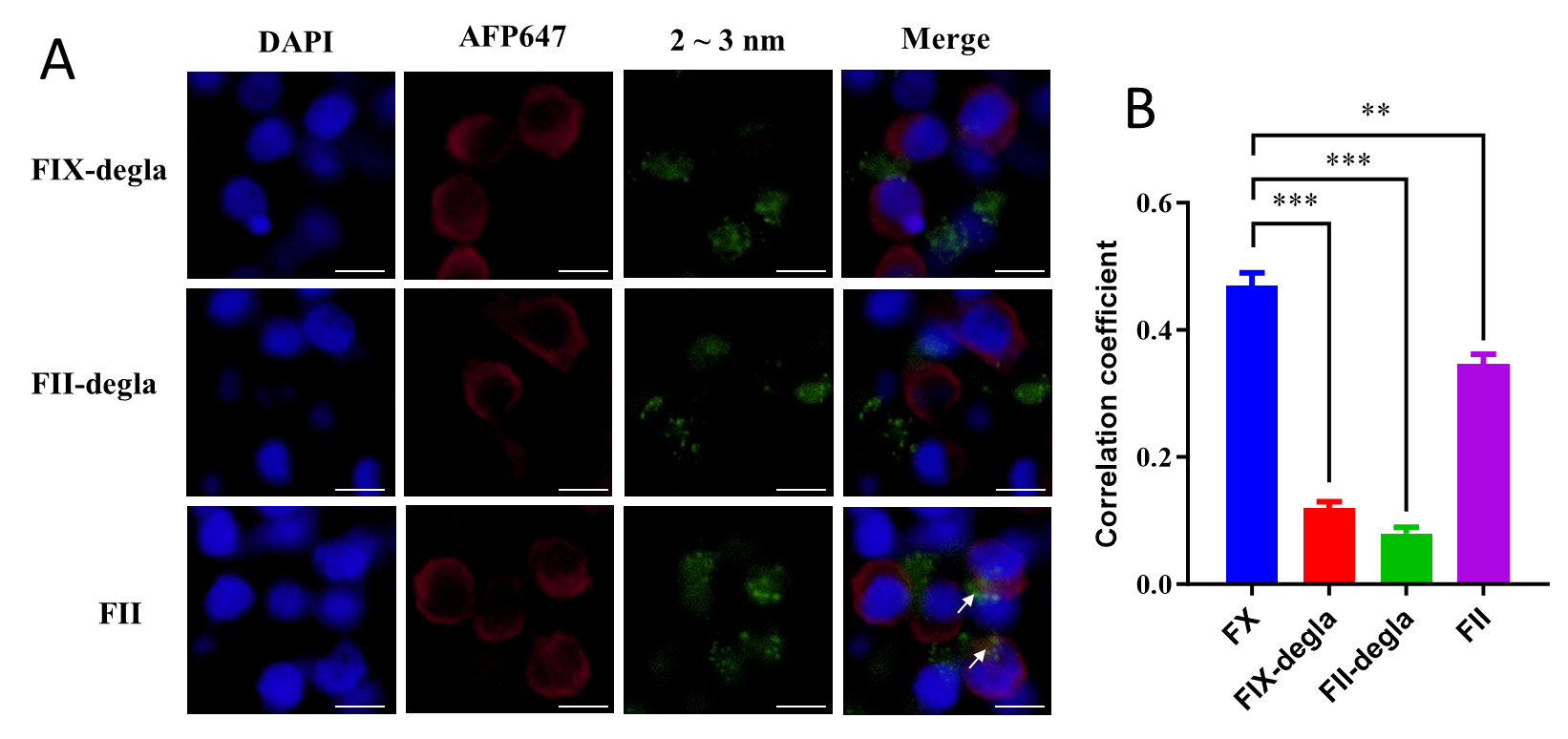


**Fig. S8.** (A) Representative fluorescence microscopy images showing colocalization between GNPs and VKD coagulation proteins. Cell nuclei counterstained with DAPI (blue), while VKD coagulation factors were labeled with Alexa Fluor Plus 647 (AFP647)-conjugated polyclonal antibody (red). White arrows indicate area of colocalization. The scale bar represents 10 μm. (B) Quantitative correlation analysis of fluorescence colocalization between GNPs and indicated VKD coagulation proteins. ***p* < 0.01, ****p* < 0.001.


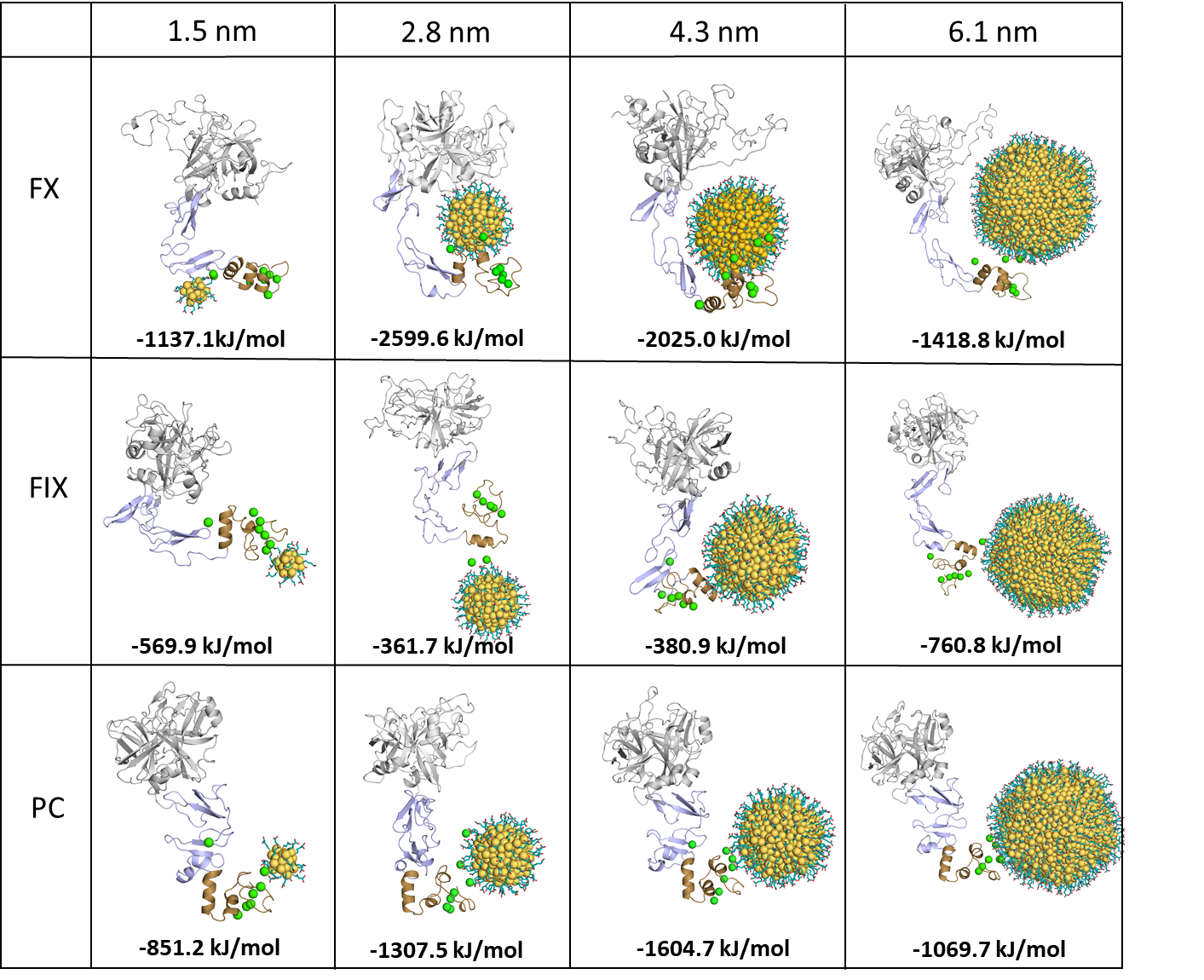


**Fig. S9.** Visual representations of the stable binding conformations of GNPs with various VKD coagulation proteins, with interacting energy displayed beneath each snapshot.


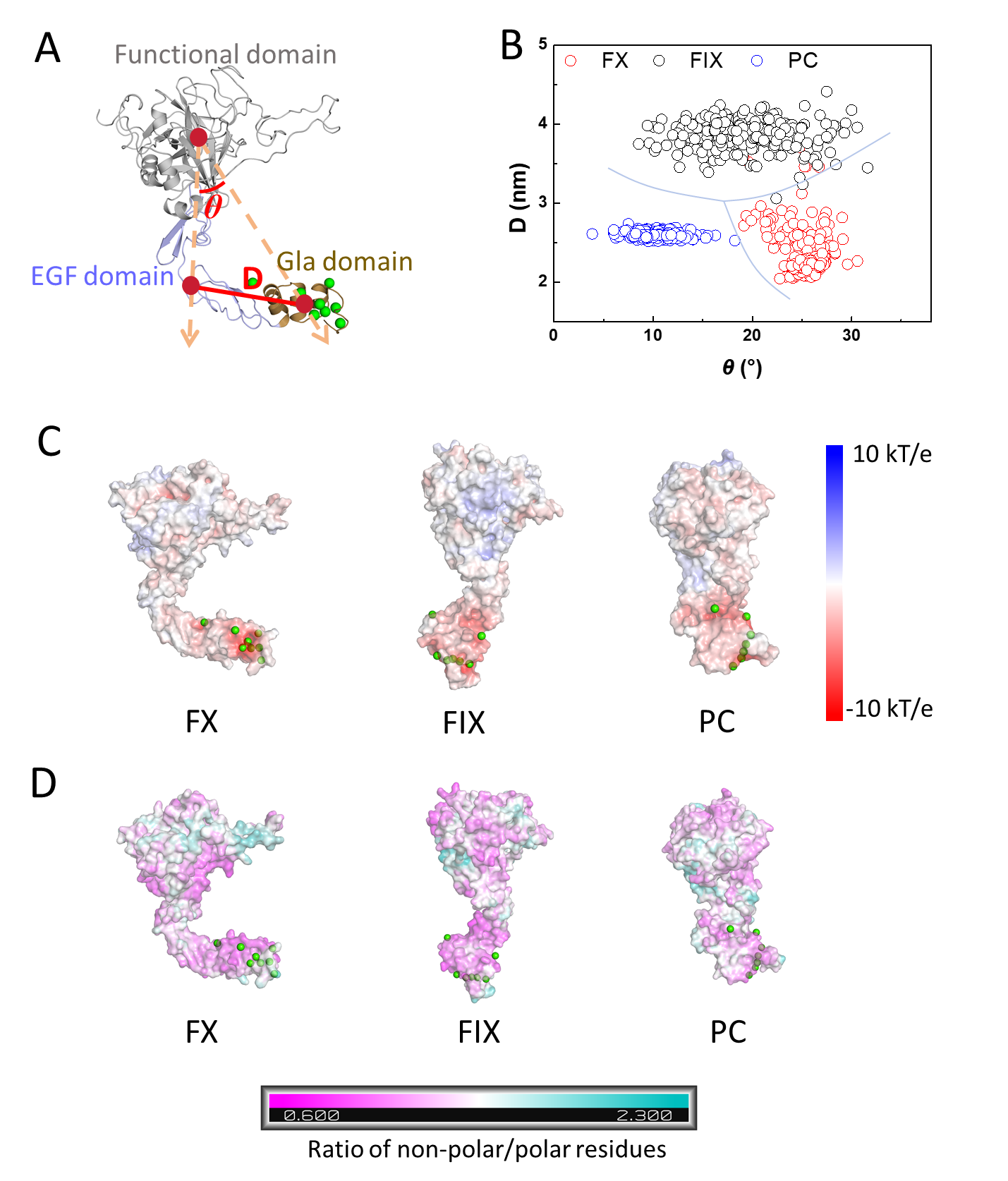


**Fig. S10.** (A) Definition of two indicators for describing the conformations of VKD coagulation proteins. (B) Analysis of the structural differences among VKD coagulation proteins derived from 200 ns molecular dynamics simulations. (C-D) Surface potential (C) and hydrophilic/hydrophobic residues (D) distribution of FX, FIX and PC.


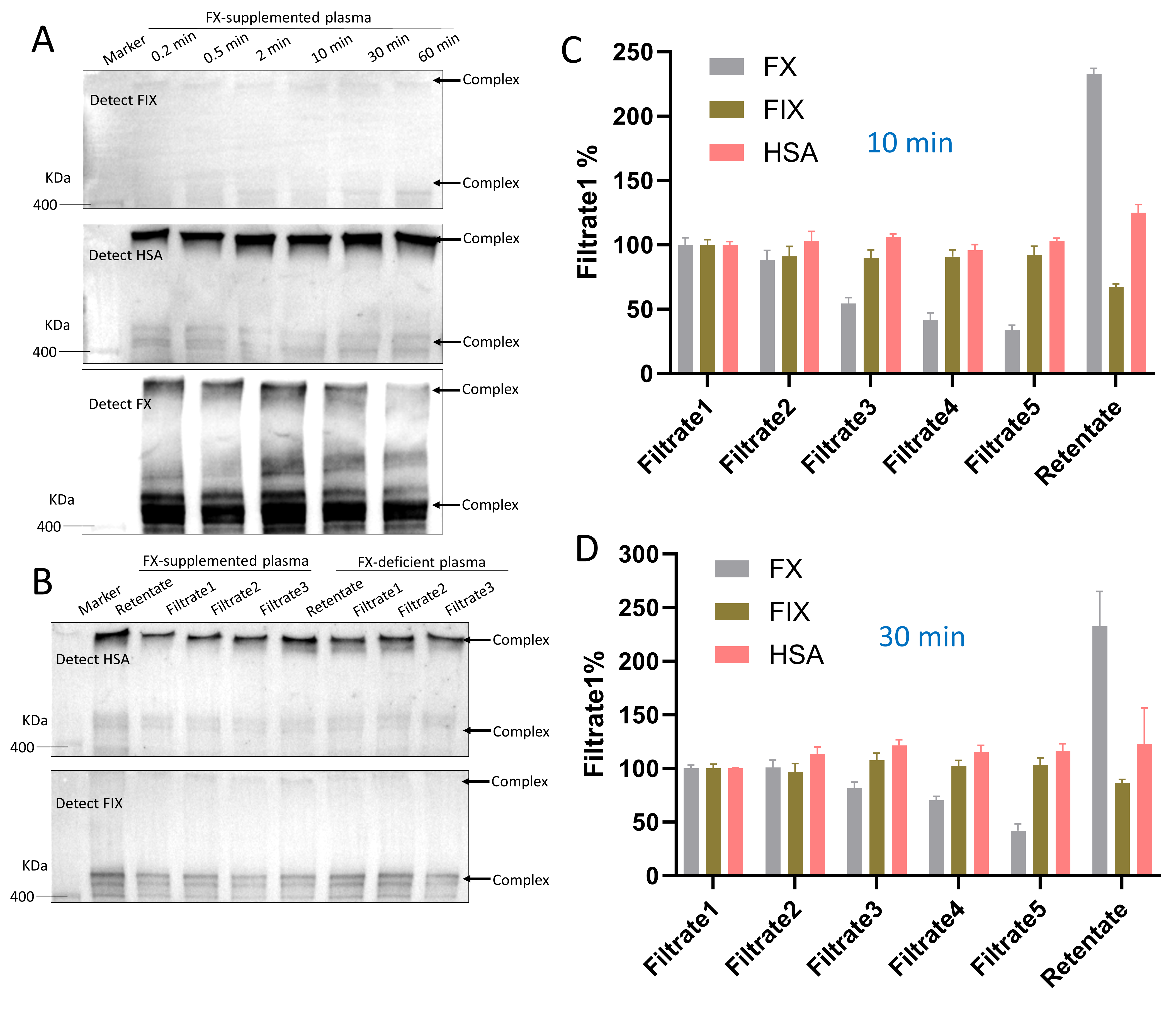


**Fig. S11.** (A) Time-course Western blot analysis demonstrating the association of various plasma proteins with GNP complexes. (B) Western blot detection of HSA and FIX in the GNP-protein complexes. The experimental procedures were identical to those in Fig. 3D, except that different proteins were probed, including HSA (top) and FIX (bottom). Arrows indicate the position of the complex as identified in Fig. 3D. (C-D) Relative concentrations of plasma proteins, including FX, FIX, and HSA, in sequential wash buffers and the final retentate after 10- (C) and 30-min (D) incubations, as determined by ELISA assays.


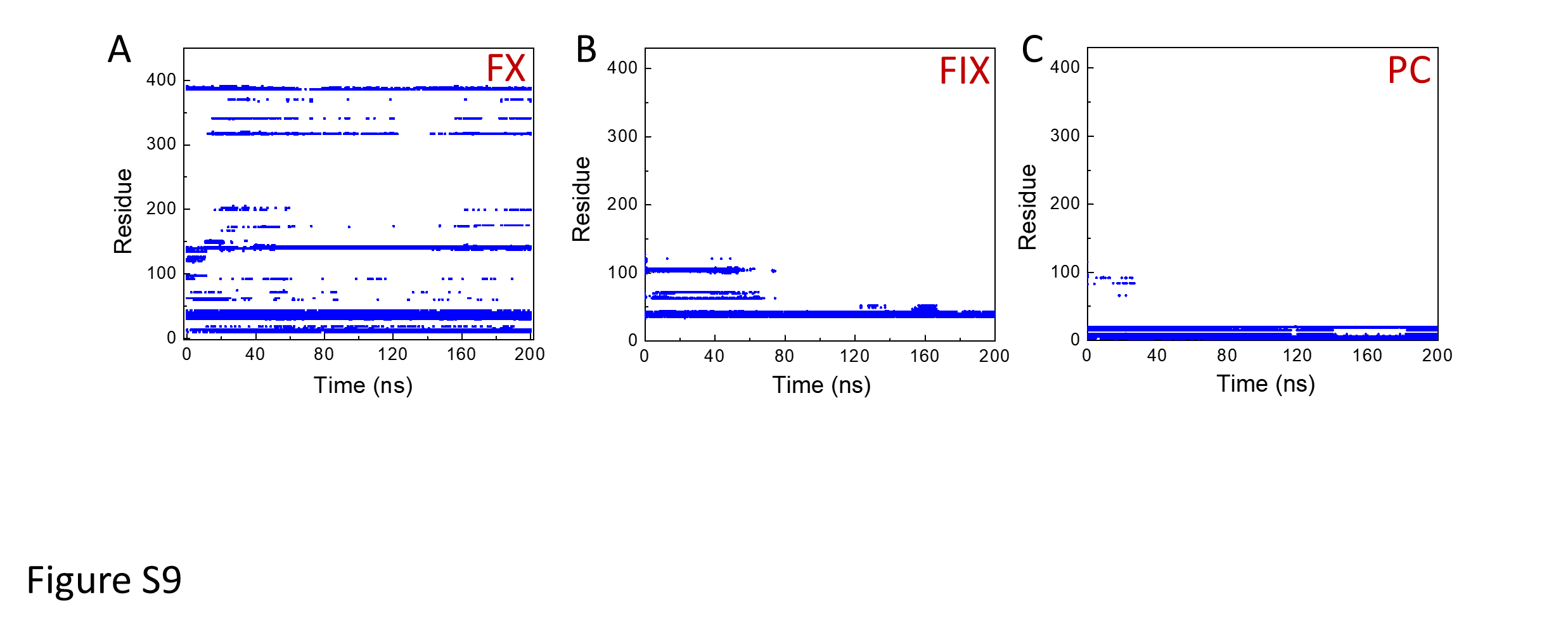


**Fig. S12.** Time evolutions of the contact distributions of amino acid residues with 2.8 nm GNP.


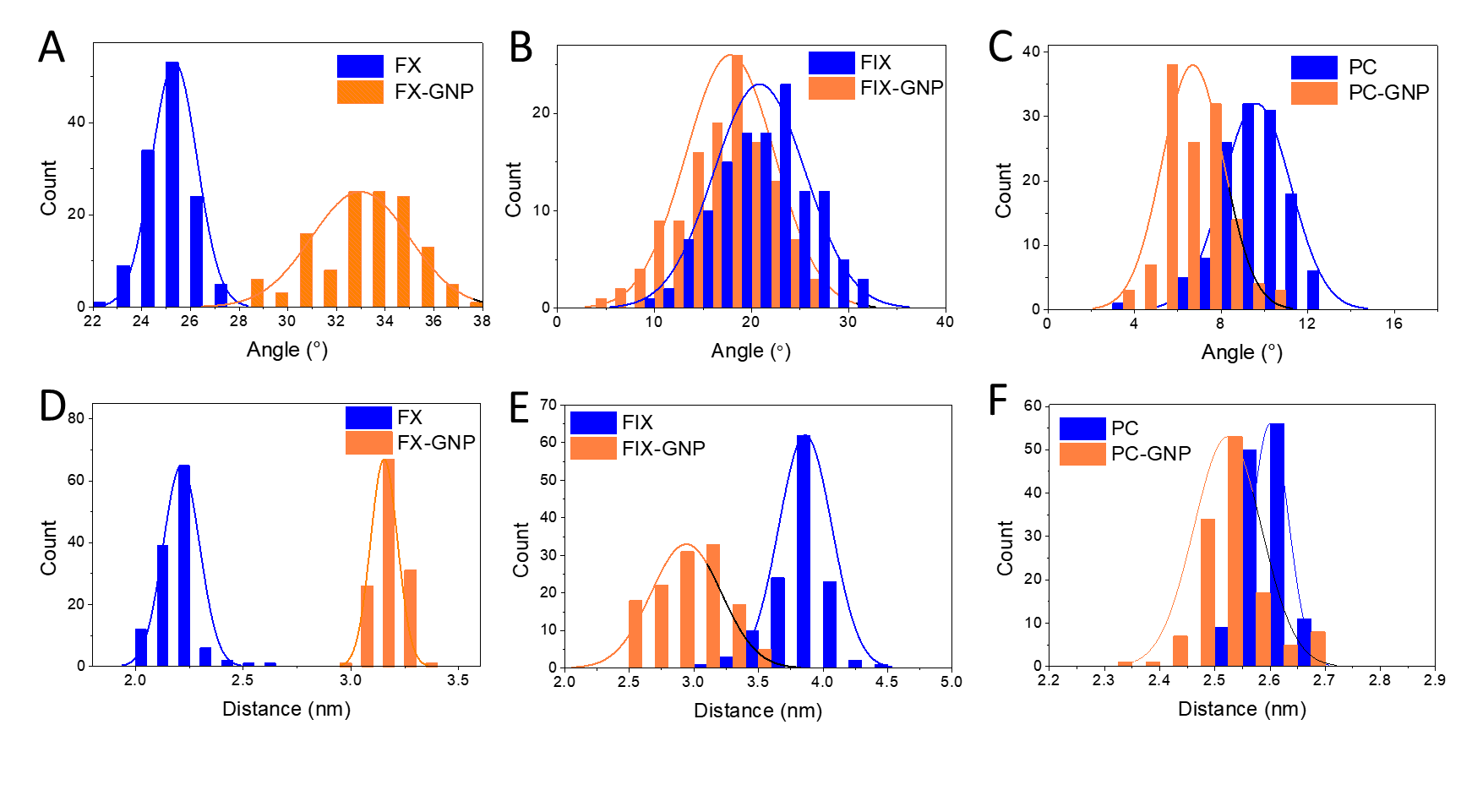


**Fig. S13.** Distribution of the defined angle (A-C) and distance (D-F) in the absence and presence of 2.8 nm GNP. (A, D) FX; (B, E) FIX and (C, F) PC.


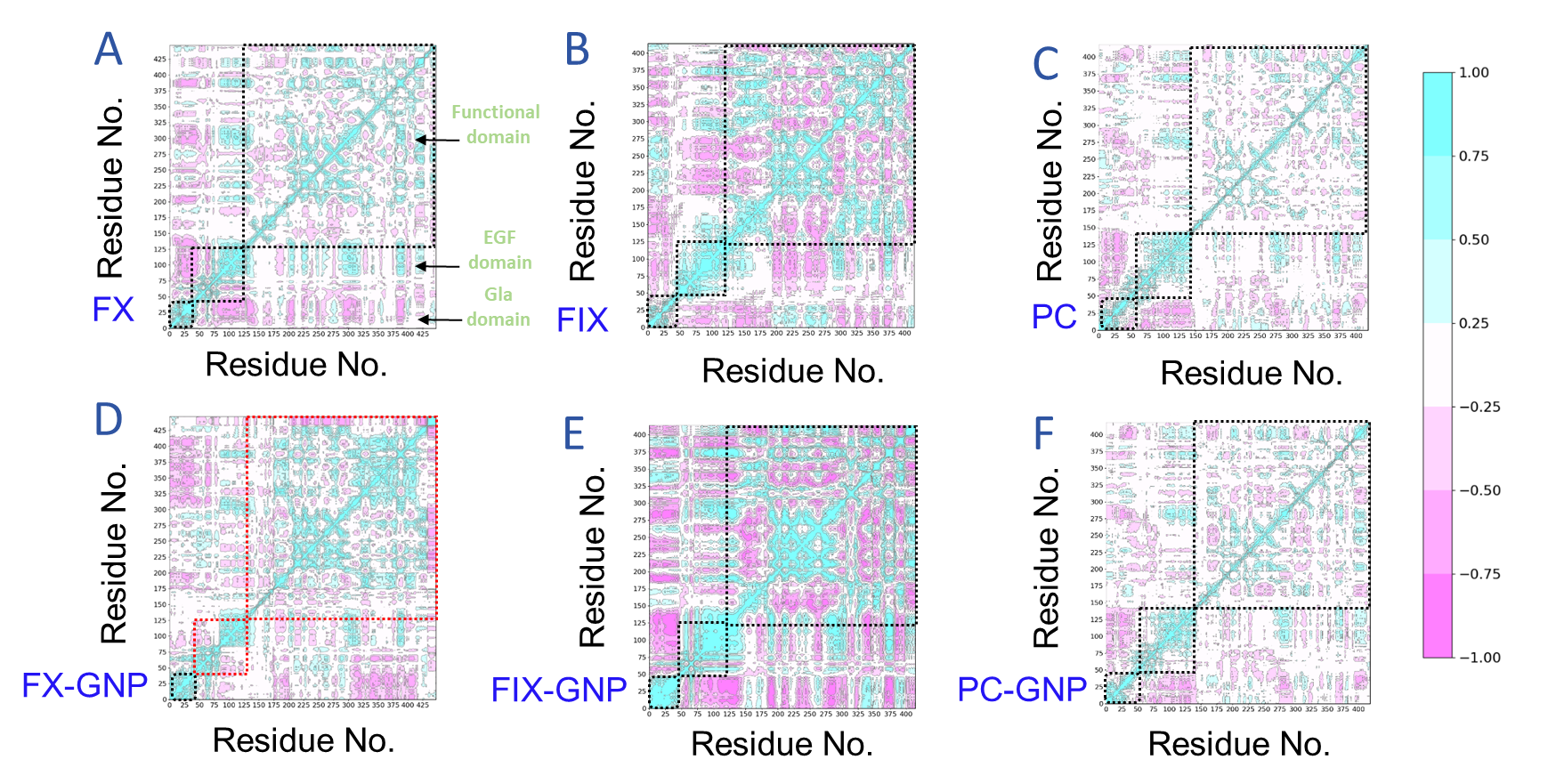


**Fig. S14.** Dynamical cross-correlation matrix of VKD coagulation proteins in the absence and presence of 2.8 nm GNP, based on final 50 ns simulations. (A, D) FX; (B, E) FIX; and (C, F) PC.


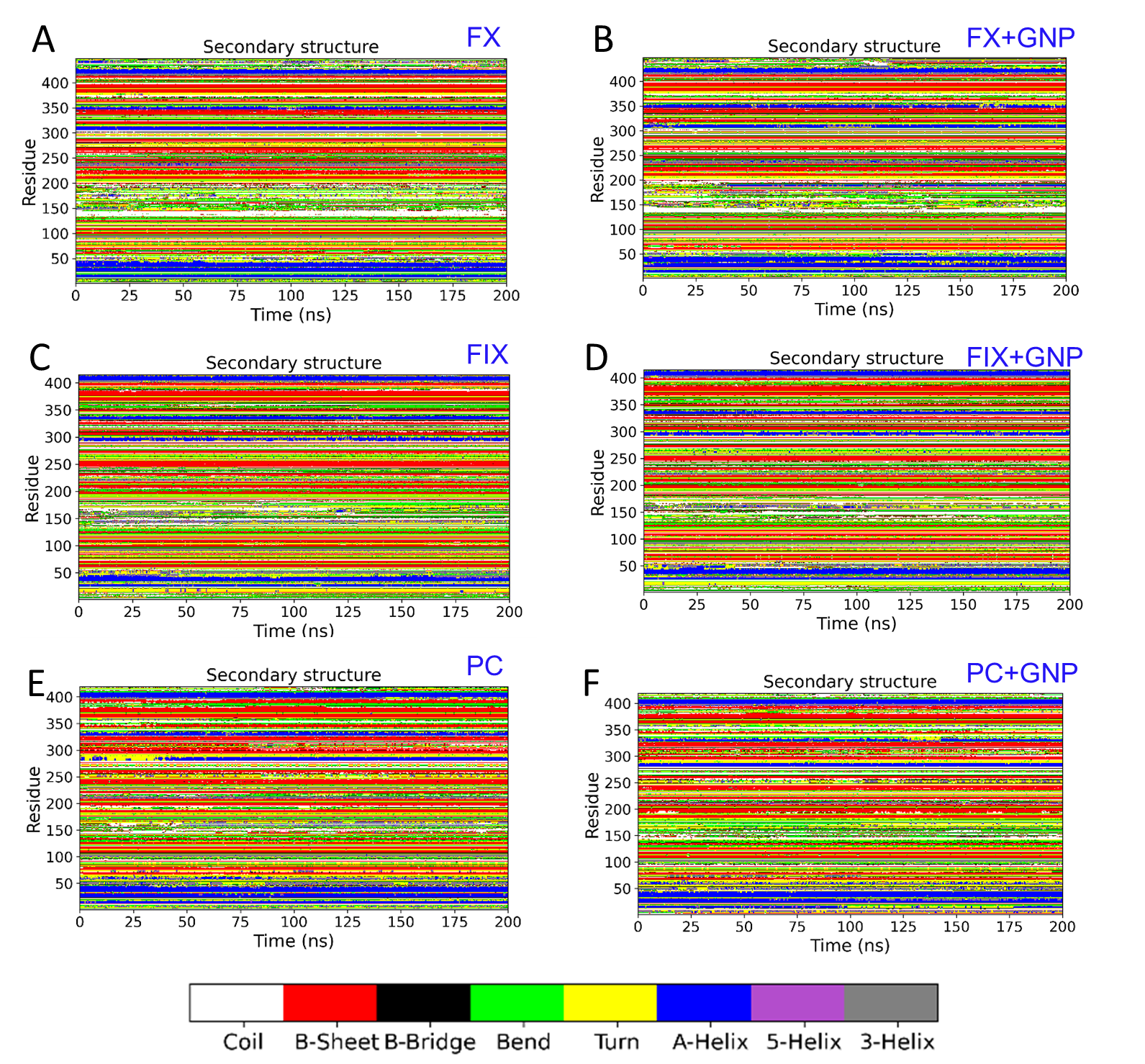


**Fig. S15.** Time evolutions of secondary structure distributions of VKD coagulation proteins in the absence and presence of 2.8 nm GNP. (A-B) FX, (C-D) FIX, (E-F) PC.


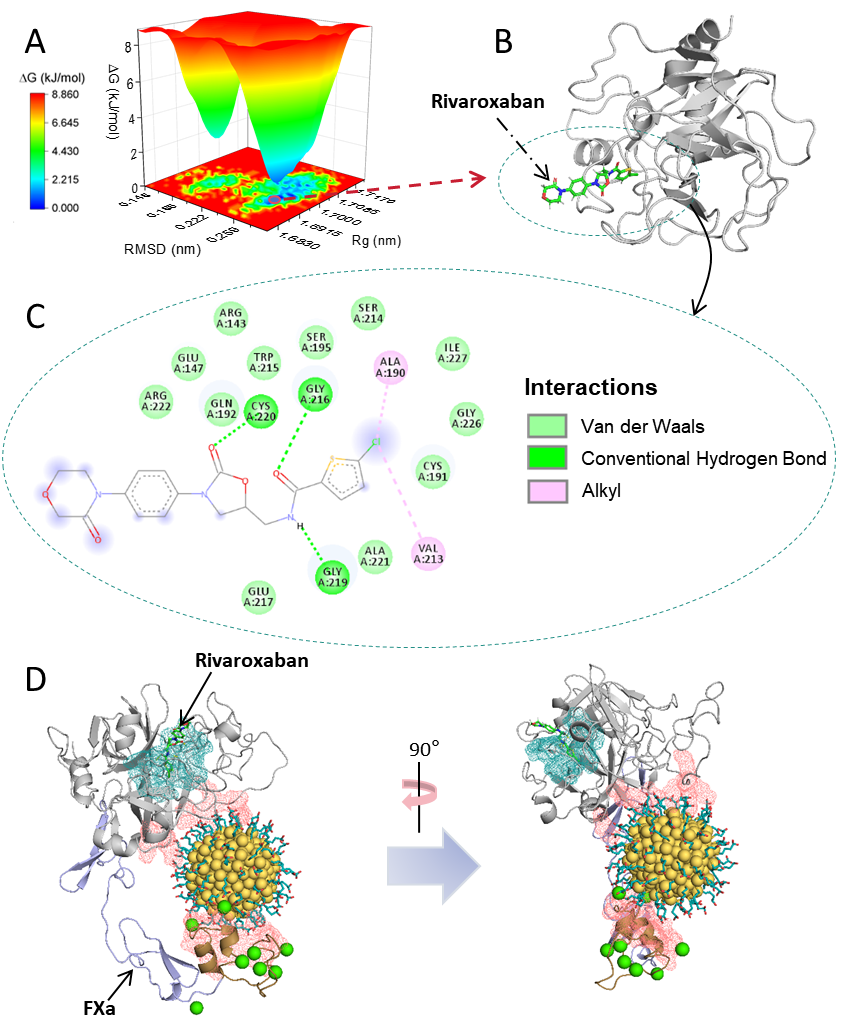


**Fig. S16.** (A) Gibbs free energy landscape obtained from a 100 ns simulation of rivaroxaban binding to the functional domain of FXa, with the initial conformation generated by molecular docking. (B) Visualization of the binding conformation. (C) Two-dimensional diagram of the binding mode. (D) Structural comparison of the rivaroxaban binding pocket (light blue) with the 2-3 nm GNP binding region (red).


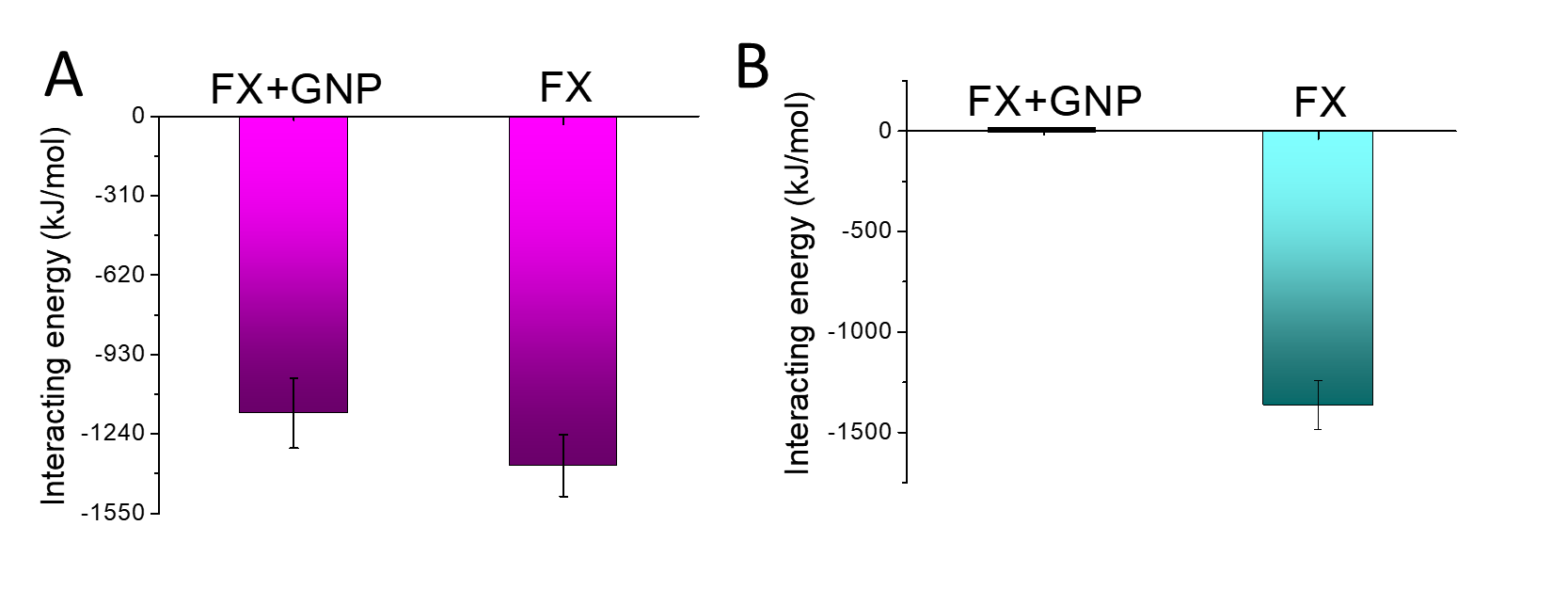


**Fig. S17.** (A) Interacting energy of lipid membrane with FX or the FX-GNP complex. (B) Interacting energy of lipid membrane with the Gla domain of FX in the absence and presence of 2.8 nm GNP.


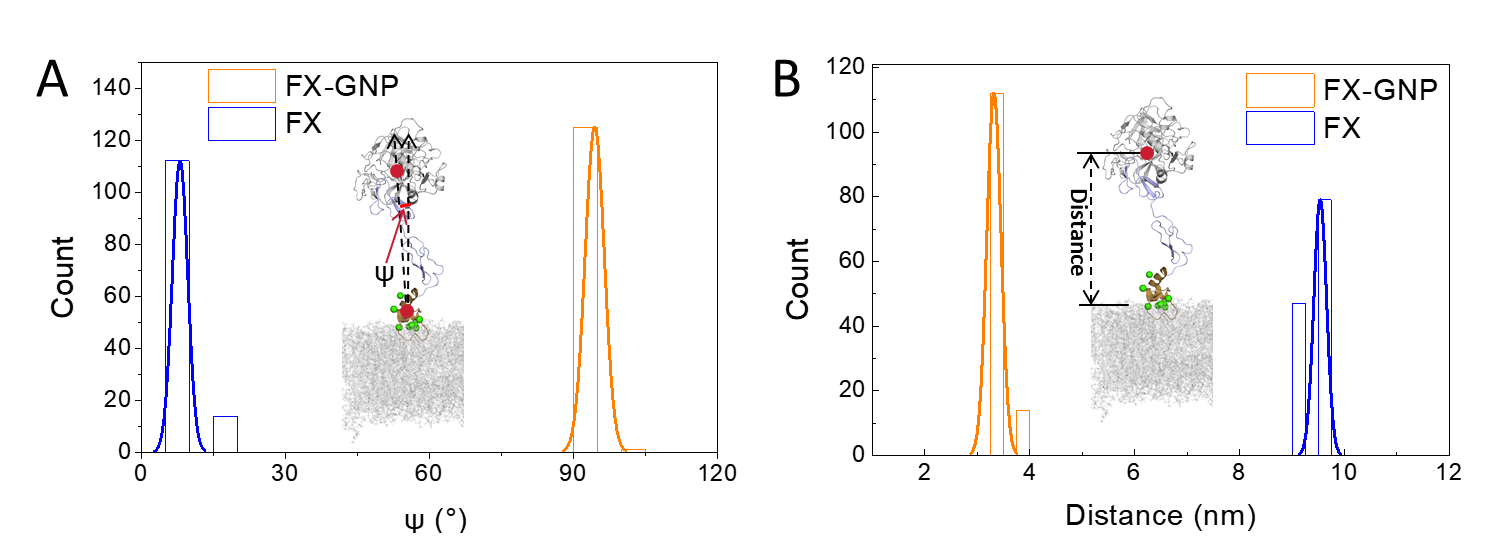


**Fig. S18.** (A) Statistical distribution of a tilt angle ψ between the vector connecting the centroids of the Gla and the functional domains with the normal vector of the phospholipid membrane. (B) Statistical distribution of the distance from functional domain centroid to the membrane surface, as defined in the inset.


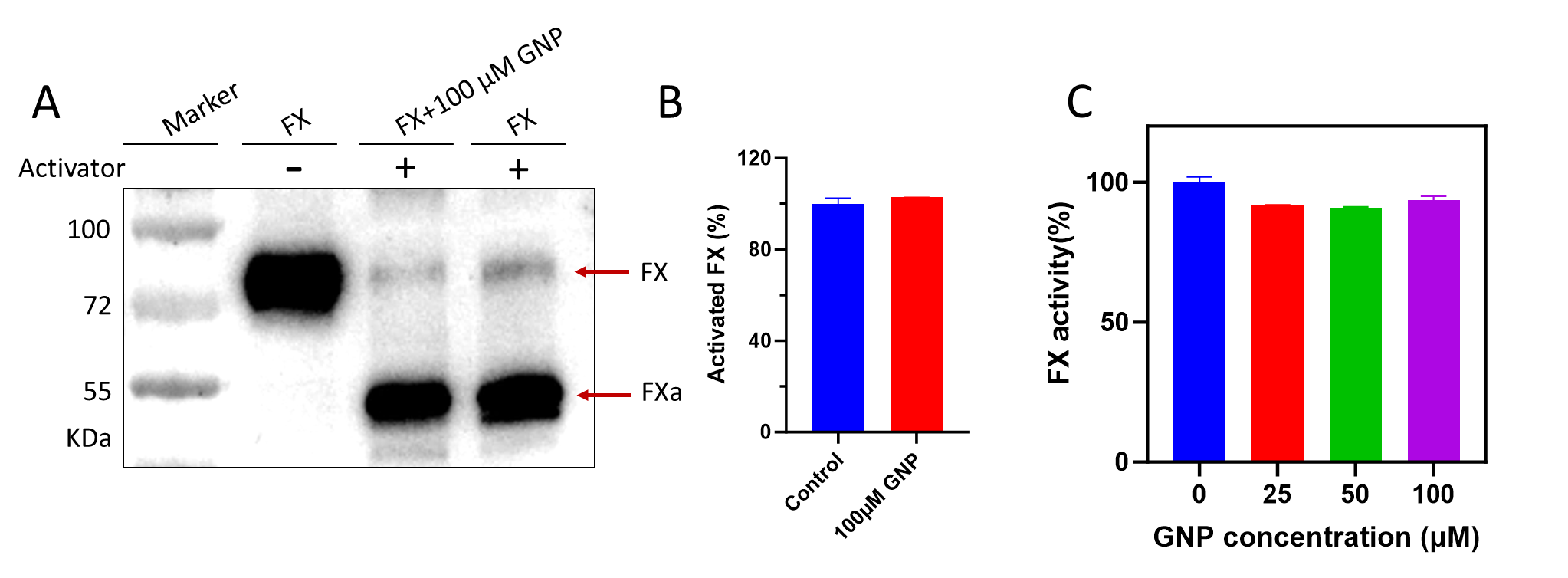


**Fig. S19.** (A) Western blot results of FX activation with or without 100 µM GNPs (> 8 nm). (B) Quantitative analysis (ImageJ-based) of FX cleavage affected by GNPs, based on grey values in (A). (C) Effect of GNPs (> 8 nm) on FX enzymatic activity using a chromogenic substrate method.


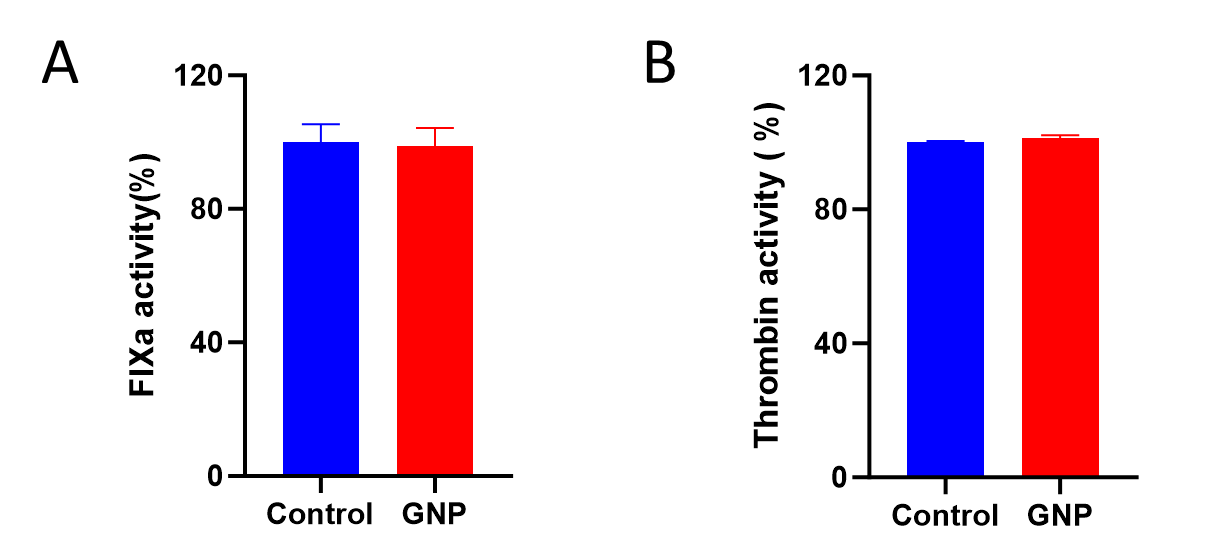


**Fig. S20.** Effect of 2-3 nm GNPs (100 µM) on the enzymatic activity of FIXa (A) and thrombin (B), measured using a chromogenic substrate assay.


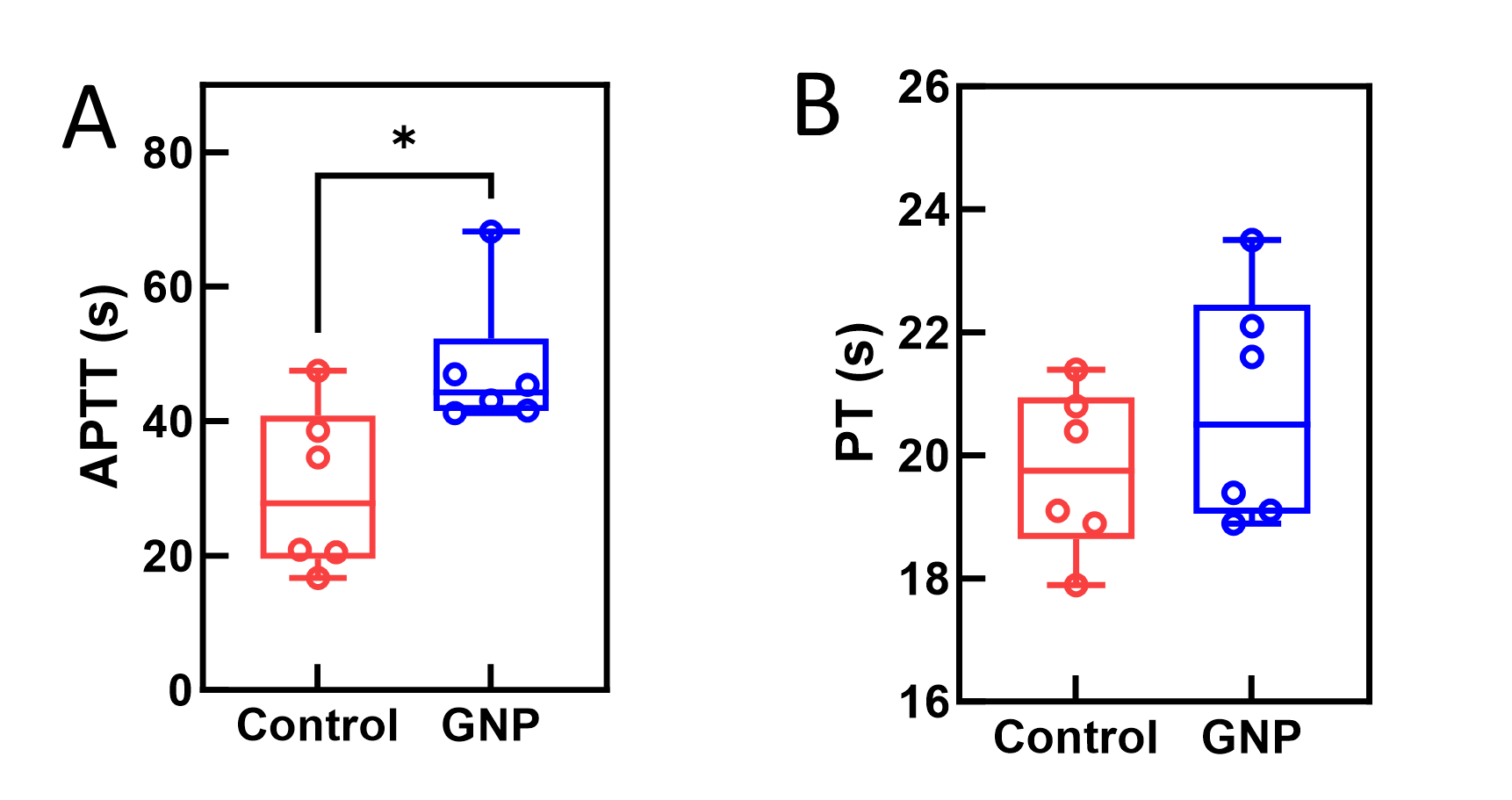


**Fig. S21.** Quantification of clotting times by APTT (A) and PT (B) assays in plasma from mice treated with GNPs or normal saline three hours after injection. **p* < 0.05.


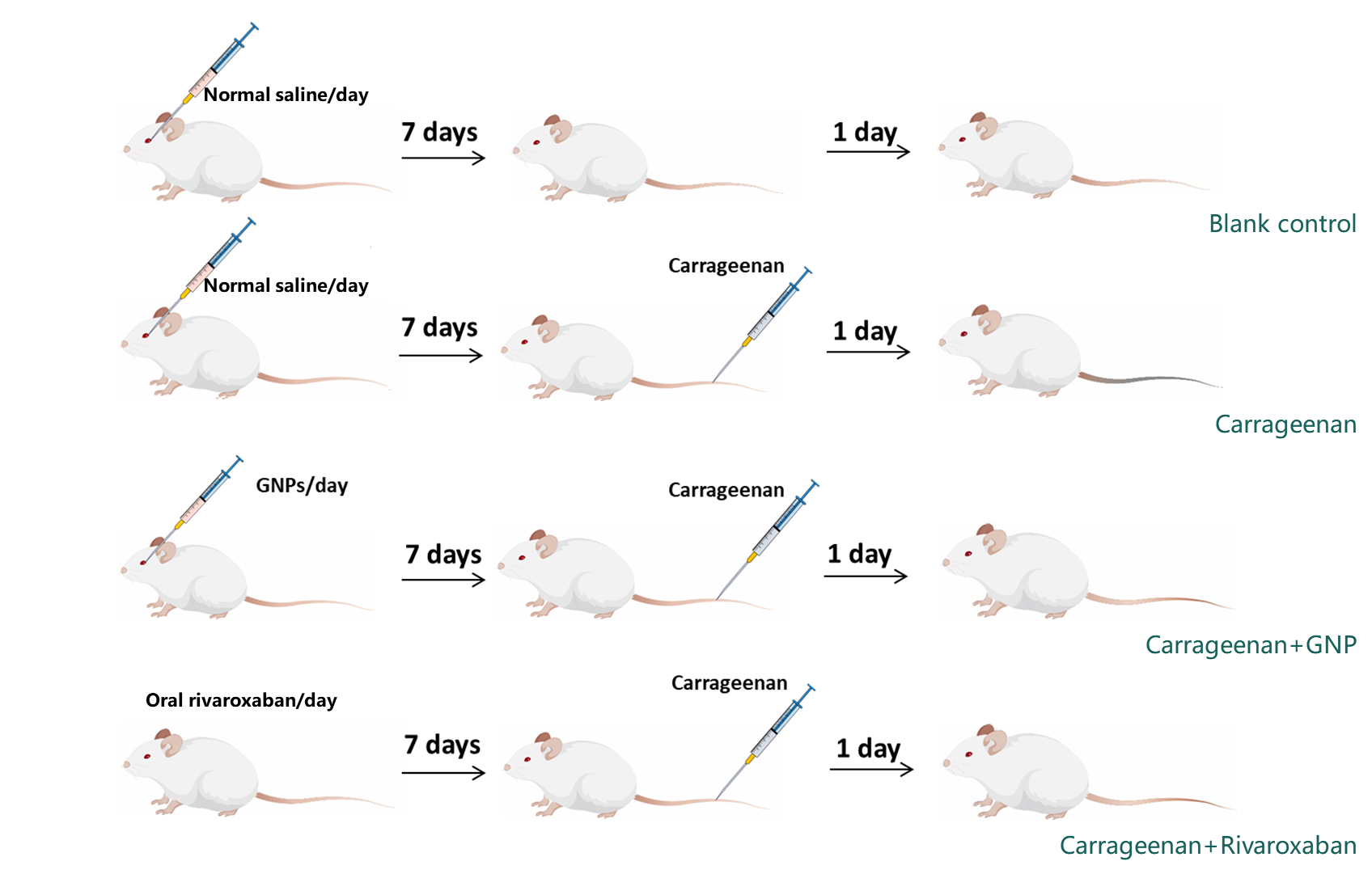


**Fig. S22.** Schematic illustration of *in vivo* experiments for evaluating venous thrombosis therapy.


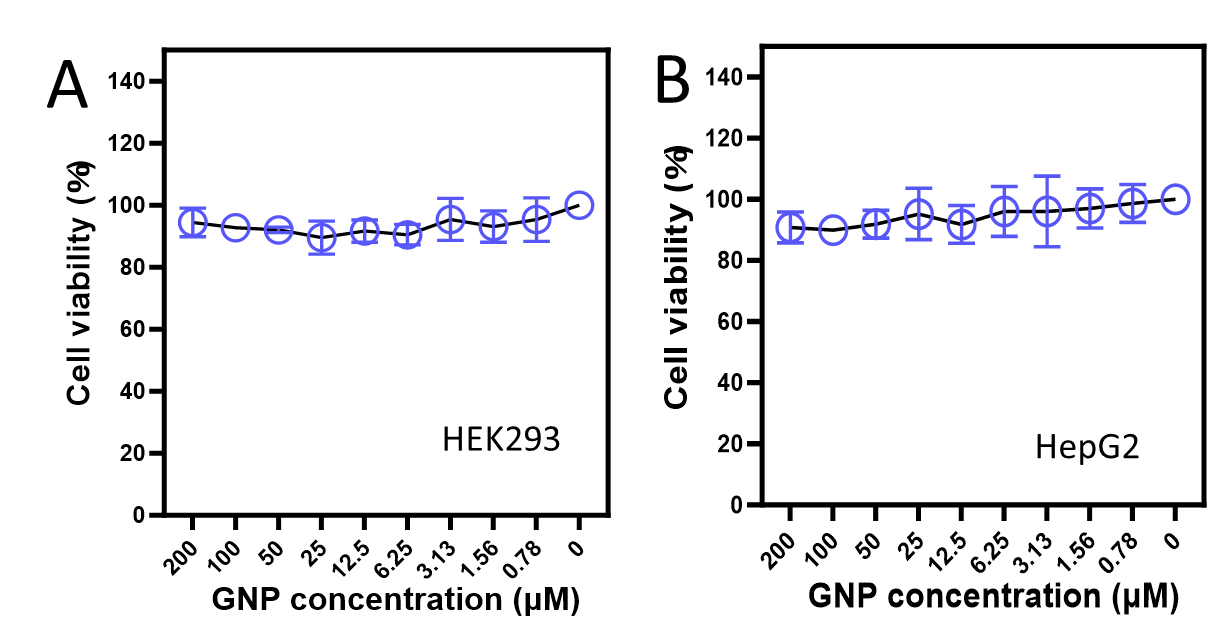


**Fig. S23.** Cytotoxicity was assessed in (A) HEK293 and (B) HepG2 cells after exposure to increasing concentrations of 2–3 nm GNPs.


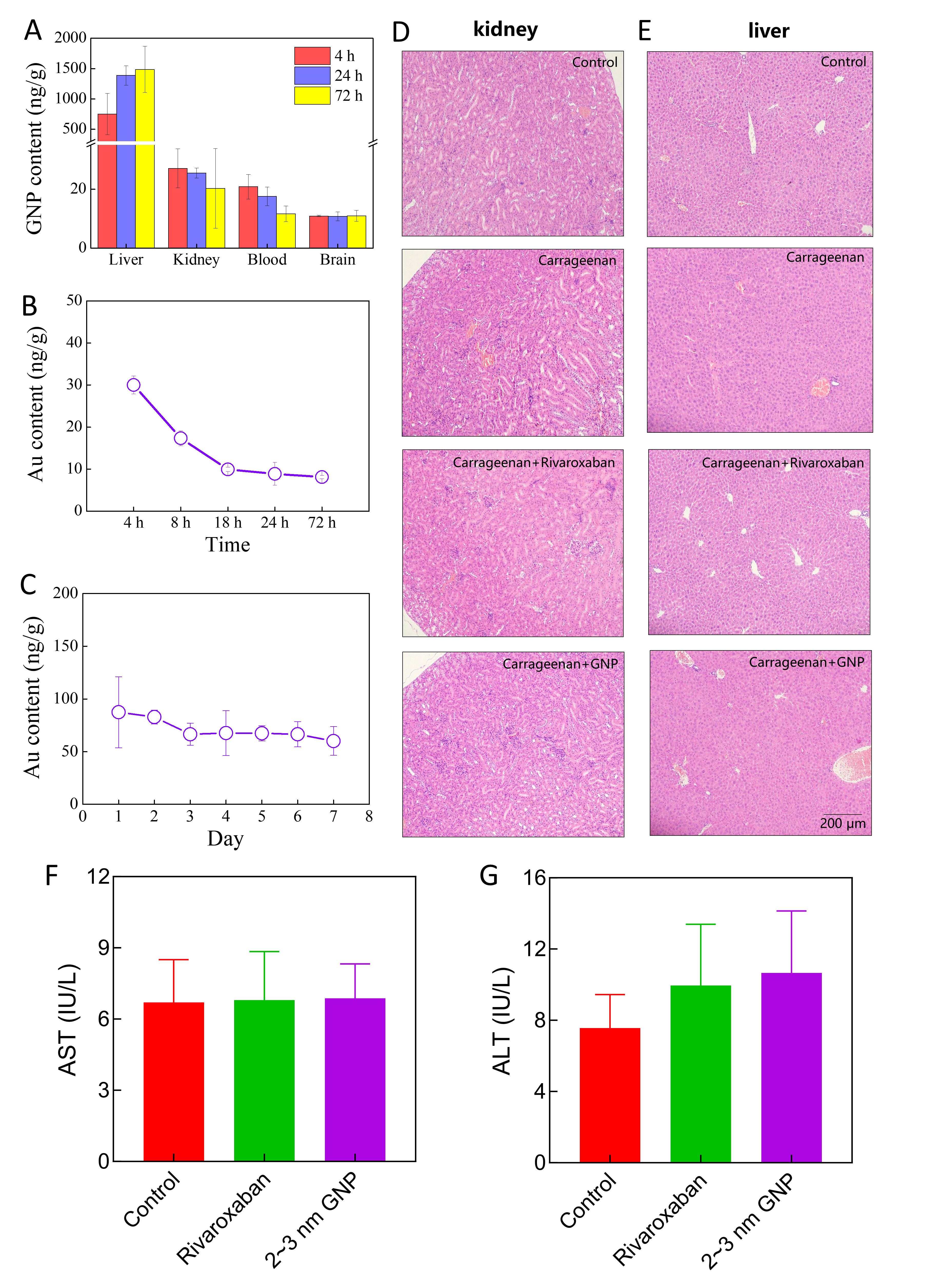


**Fig. S24.** (A) Au content in mice’ tissues after single GNP (2-3 nm) injection at various time. (B-C) Time evolution of GNP content in mice’ feces under single (B) and continuous (C) GNP injection (100 µL of 100 µM solution per day). (D-E) H&E staining images of the main organs (kidney (D) and liver (E)) after 7 days of GNPs treatments. (F-G) *In vivo* analysis of AST (F) and ALT (G) concentrations under different treatments.

**Table S1.** Thermodynamic quantities for the binding kinetics of 2-3 nm GNP interactions with FX, FIX and PC.

|  | Ka (×10^5^ M^-1^) | Kd (×10^-6^ M) | △H (kJ/mol) | △S (J/mol•K) | N |
| --- | --- | --- | --- | --- | --- |
| FX | 16.39 | 0.61 | -12.31 | 77.69 | 0.78 |
| FIX | 4.03 | 2.48 | -6.28 | 86.23 | 1.45 |
| PC | 4.00 | 2.50 | -5.81 | 87.80 | 1.98 |

**References**

1. J. Jumper, R. Evans, A. Pritzel, T. Green, M. Figurnov, O. Ronneberger, K. Tunyasuvunakool, R. Bates, A. Žídek, A. Potapenko, Highly accurate protein structure prediction with AlphaFold, Nature 596 (7873) (2021) 583-589, http://doi.org/10.1038/s41586-021-03819-2.
2. S. Jo, X. Cheng, J. Lee, S. Kim, S.J. Park, D.S. Patel, A.H. Beaven, K.I. Lee, H. Rui, S. Park, CHARMM‐GUI 10 years for biomolecular modeling and simulation, J. Comput. Chem. 38 (15) (2017) 1114-1124, http://doi.org/10.1002/jcc.24660.
3. G.M. Morris, R. Huey, W. Lindstrom, M.F. Sanner, R.K. Belew, D.S. Goodsell, A.J. Olson, AutoDock4 and AutoDockTools4: Automated docking with selective receptor flexibility, J. Comput. Chem. 30 (16) (2009) 2785-2791, http://doi.org/10.1002/jcc.21256.
4. O. Trott, A.J. Olson, AutoDock Vina: improving the speed and accuracy of docking with a new scoring function, efficient optimization, and multithreading, J. Comput. Chem. 31 (2) (2010) 455-461, http://doi.org/10.1002/jcc.21334.
5. D.S.D. Van, E. Lindahl, B. Hess, G. Groenhof, A.E. Mark, H.J. Berendsen, GROMACS: fast, flexible, and free, J. Comput. Chem. 26 (16) (2005) 1701, http://doi.org/10.1002/jcc.20291.
6. W.L. DeLano, Pymol: An open-source molecular graphics tool, CCP4 Newsl. Protein Crystallogr 40 (1) (2002) 82-92, https://legacy.ccp4.ac.uk/newsletters/ newsletter40/11_pymol.html.
7. W.K. Ko, D.N Heo, H.J Moon, S.J. Lee, M.S. Bae, J.B. Lee, I.C Sun, H.B. Jeon, H.K. Park, I.K Kwon. The effect of gold nanoparticle size on osteogenic differentiation of adipose-derived stem cells, J. Colloid Interface Sci. 438 (2015) 68-76, https://doi.org/10.1016/j.jcis.2014.08.058.
